# Characteristics of gut microbiota dysbiosis in patients with depression: a visual analysis of knowledge maps

**DOI:** 10.3389/fmicb.2026.1937281

**Published:** 2026-09-07

**Authors:** Chenggui Xu, Shouyao Zhang, Yong Liao, Exian Shan, Pengbin Zhao, Ruilu Liu, Jiayu Wang, Minjie Yin, Xiantao Tai, Xinghe Zhang

**Affiliations:** 1 The Second Clinical Medical School, Yunnan University of Chinese Medicine, Kunming, China; 2 The First Clinical Medical School, Yunnan University of Chinese Medicine, Kunming, China

**Keywords:** bacteria, depression, gut microbiota, knowledge map, review

## Abstract

**Background:**

Patients with depression exhibit significant gut microbiota dysbiosis; however, visualized studies into dysbiosis-related microbial signatures in depression remain limited. To identify characteristic differential genera in depressed individuals, this study adopted a knowledge graph–based visualization analysis to clarify the associations between intestinal microbial dysbiosis and depression.

**Methods:**

A search strategy was constructed and literature searches were conducted in 7 databases, including Web of Science, PubMed, Scopus, Cochrane Library, CNKI, Wanfang Database and VIP Net (VIP). The search cutoff date was April 1, 2026. The included literature was strictly screened according to the PRISMA guidelines. EndNote 25 was used to sort and trace the literature meeting the inclusion criteria. GRADE system, Cochrane ROB2.0, and JBI scale were used for quality evaluation and evidence grading of RCTs and cross-sectional studies.

**Results:**

A total of 159 valid studies from 21 countries were ultimately included, involving 25310 patients with depression. The bacterial community taxa predominantly consisted of *Bacillota, Bacteroidota*, and *Actinomycetota*, among which *Bacillota* was overwhelmingly dominant. *Ascomycota* predominated within the fungal community. For bacteria, 73 genera including *Faecalibacterium* and *Butyricicoccus*, alongside 66 species such as *Levilactobacillus brevis* and *Bifidobacterium animalis*, exhibited depleted abundances. Such reductions were linked to diminished production of butyrate-producing commensal beneficial bacteria. 43 genera (e.g., *Escherichia* and *Coprobacillus*) and 21 species (including *Escherichia-Shigella* and *Clostridium bolteae*) belonging to pro-inflammatory and pathogenic microbes showed elevated levels. 25 genera represented bidirectionally altered taxa with both increased and decreased abundances, exemplified by *Bifidobacterium* and *Bacteroides*. Regarding fungi, 16 taxa including *Aureobasidium* and *Saccharomyces cerevisiae boulardii* were less abundant, whereas 10 taxa such as *Candida* and *Saccharomyces* had higher abundances.

**Conclusion:**

Dysbiosis in patients with depression was characterized by markedly decreased abundances of protective microbes including *Faecalibacterium* and *Aureobasidium*, accompanied by concurrent enrichment of pathogenic bacteria such as *Escherichia* and *Candida* in the gut. Taxa exhibiting bidirectional abundance shifts may be affected by confounders including age and gender, which requires further high quality clinical validation. Targeted modulation of disrupted microbiota, development of strain-specific biologics, and species-level profiling of microbial variations will enhance the therapeutic benefits of microecological preparations for depression adjuvant treatment.

## 1 Introduction

Depression ranks among the most prevalent mental disorders, with its incidence rising annually. According to the World Health Organization (WHO) statistics, approximately 5% of adults globally are affected by depression, and major depressive disorder has emerged as a leading contributor to the loss of disability-adjusted life years (Gao et al., 2023). This condition not only significantly impairs patients’ social functioning and quality of life but also imposes a substantial economic burden on families and society (Culpepper et al., 2024). Nonetheless, numerous challenges persist in the treatment of depression, including a slow onset of effects, low remission rates, and pronounced side effects (Chen L. C. et al., 2020). Approximately one-third of patients exhibit poor responses to an adequate course of antidepressant treatment, ultimately developing treatment-resistant depression (Smith, 2014). This situation underscores the urgent need for novel therapeutic targets and intervention strategies.

In recent years, the role of gut microbiota in the pathogenesis of mental disorders has received extensive attention. The human intestinal tract is home to trillions of microorganisms, including bacteria, viruses and fungi, which together form the human microbiome (Sorboni et al., 2022). Research has found that gut microbiota can form bidirectional communication with the central nervous system through multiple pathways such as vagus nerve conduction, immune signal regulation, tryptophan metabolism, and synthesis of neuroactive substances, namely the “gut-brain axis” (Goel et al., 2025; Yassin et al., 2025; Chen et al., 2024). Dysfunction of this axis is considered closely associated with the onset and persistence of depressive symptoms. Systematic reviews and meta-analyses have revealed that patients with depression exhibit an elevated relative abundance of pro-inflammatory taxa and reduced abundance of butyrate-producing genera within the gut microbiota (Knudsen et al., 2021). Meanwhile, dysregulation of taxa such as *Alloprevotella*, which is positively correlated with the kynurenine-to-tryptophan ratio, has also been observed (Yan et al., 2026), implying complex crosstalk among immune-inflammatory responses, hormones and gut microbiota. Further clinical studies have confirmed significant compositional differences in the gut microbiota between depressed patients and healthy controls. Enrichment of *Collinsella* and *Eggerthella* has been reported in patients with depression, which may contribute to disease pathogenesis (Zhong et al., 2022; Guo et al., 2024). Microbe-targeted interventions (including probiotics, prebiotics and synbiotics) exert certain beneficial effects on alleviating depressive symptoms (Alli et al., 2022; Asad et al., 2025). Nevertheless, their efficacy is modulated by geographical region, patient comorbidities, intervention duration and other confounding factors, leading to substantial heterogeneity across studies (Pan et al., 2025). Collectively, these findings lend strong support to the gut-brain axis hypothesis and shed light on the development of microbiota-based adjuvant therapeutic strategies for depression.

Although accumulating evidence has confirmed the close association between gut microbiota and depression, existing studies exhibit substantial heterogeneity and lack systematic integration. This situation makes it difficult to distinguish consistently reported core microbial biomarkers from insufficient or contradictory microbial signatures across independent studies, thereby hindering the translational application of microbiota-related findings in clinical practice. To address these gaps, the present study comprehensively synthesizes current evidence regarding depression-associated microbial alterations and aims to: (1) identify the most robust, high-level microbial signatures that are stably correlated with depression; (2) elaborate inconsistent and controversial findings in the current literature; (3) provide prospective recommendations for the application of probiotics as an adjuvant therapy for depression.

## 2 Methods

Through a systematic retrieval and screening process of all randomized controlled trials, cross-sectional studies, systematic reviews, meta-analyses, and case reports pertaining to the association between gut microbiota and depression since the inception of each database, we ultimately retained clinical research literature that met the inclusion criteria. Following a risk and quality evaluation of the literature, we extracted microbiota information that exhibited significant differences across the reports. This information was then summarized and organized according to the hierarchical structure of international standard bacterial taxonomy. Taxonomic notes and name checks for all flora are unified in NCBI (National Center for Biotechnology Information)^1^ and GTDB (GenomeTaxonomy Database).^2^ Both were consulted, with GTDB was used as the main classification standard, NCBI as supplementary verification, to achieve accurate classification and positioning from kingdom, phylum, class, order, family, genus to species and subspecies). Meanwhile, a comprehensive evidence-grading evaluation system was constructed. Based on this system, we assessed the association strength and evidence quality for each microbial taxon linked to depression. Origin 2026 was employed to construct a knowledge graph of depression related microbiota: a sunburst diagram was plotted according to the taxonomic hierarchy from kingdom, phylum, class, order, family, genus to species; stacked bar charts were generated to reflect alterations in microbial relative abundance together with corresponding evidence grades; and a sankey diagram was used to intuitively illustrate the top-down taxonomic affiliation and community structural flow patterns of gut microbiota. This study aims to reveal the taxonomic characteristics and abundance variation patterns of gut microbiota in patients with depression, identify high-confidence key microbial biomarkers, and provide evidence-based references for the clinical application of biological agents.

### 2.1 Search strategy

#### 2.1.1 Search database and date

This study adopted a comprehensive retrieval strategy across multiple databases covering mainstream Chinese and English language academic resources. The English databases included Web of Science, PubMed, Scopus and Cochrane Library; the Chinese databases consisted of CNKI, Wanfang Database, and VIP. Literature retrieval spanned from database inception to April 1, 2026. According to research designs and evidence sources, publications were divided into two categories: Primary Studies including randomized controlled trials (RCTs), cross-sectional studies, case analyses and case series; Secondary Studies comprising systematic reviews/meta-analyses, traditional narrative reviews, and clinical practice guidelines (CPGs). Two investigators independently performed literature searching and cross-checked outcomes to avoid missing and incorrect retrievals. Discrepancies were settled by a third-party researcher.

With depression as the disease topic, and gut microbiota, intestinal microorganisms, probiotics, bacteria, and fungi as microbial-related topics, search strategies were constructed following the specifications of each database (Table 1).

**TABLE 1:** Search terms.

		Arch terms
Disease	D1	Depression
	D2	MDD
	D3	Depressive disorder
	D4	Depressed
	D5	Major depressive disorder
Bacteria	B1	Intestinal flora
	B2	Intestinal bacteria
	B3	Enterobacteria
	B4	Gut microbiota
	B5	Microorganism
	B6	Microbiota
	B7	Microbe
	B8	Germ
	B9	Fungus
	B10	Fungi
	B11	Mycoflora
	B12	Bacterial
	B13	Bacteria
	B14	Bacterium
	B15	Bacterial colony
	B16	Grisea
	B17	Probiotics
	B18	Mold
	B19	Flora

Constructing search formula: (D1 or D2 or D3 or D4 or D5) and (B1 or B2 or B3 or B4 or B5 or B6 or B7 or B8 or B9 or B10 or B11 or B12 or B13 or B14 or B15 or B16 or B17 or B18 or B19).

### 2.2 Inclusion and exclusion criteria

#### 2.2.1 Inclusion criteria

(1) Eligible study types were original studies, including randomized controlled trials, cross-sectional studies, and case reports.
(2) Studies focused on the correlation between depression and human gut microbiota, including clinical and clinical-basic research.
(3) Study subjects were humans.
(4) Studies that employed high-throughput sequencing technologies (e.g., 16S rRNA gene sequencing and metagenomic sequencing) or other molecular techniques capable of quantitatively profiling microbial community composition, thereby providing original sequencing reads and relative abundance data for the gut microbiota.
(5) All included clinical studies strictly conformed to the diagnostic criteria for depression. Participants were diagnosed with depression based on the Diagnostic and Statistical Manual of Mental Disorders, Fifth Edition (DSM-5), and the severity of depressive symptoms was assessed using the 17-item Hamilton Depression Rating Scale (HAMD-17). Participants with HAMD-17 scores ≥ 8 points met the diagnostic threshold for depressive disorders.

#### 2.2.2 Exclusion criteria

(1) Animal experiments and cell experiments.
(2) Studies without detection of gut microbiota, in which only therapeutic effects were observed.
(3) Studies with insufficient original data that failed to extract valid microbial abundance and taxonomic information.
(4) Duplicate publications, conference abstracts, systematic reviews, popular science articles, and unpublished research reports.
(5) Studies without available full-text manuscripts.
(6) Studies on depression comorbid with other severe physical or psychiatric disorders (e.g., post-stroke depression) were excluded.

### 2.3 Literature screening and data extraction

Literature screening strictly followed the PRISMA process (Liberati et al., 2009)^3^ for literature screening. First, EndNote 2025 was applied to remove duplicate records. Duplicates were further manually removed by screening titles and abstracts; obviously irrelevant records, animal-based studies, and retracted articles were excluded at this stage. Second, full-text screening was performed. Records without accessible full texts, studies with unavailable key data, and those inconsistent with the research theme were excluded, and the remaining records were categorized by study type. For secondary literature, relevant conclusions were traced back to their primary source studies. The traced original studies were further assessed against the predefined inclusion criteria for eligibility. The screening process was performed by one reviewer and supervised by a second reviewer. Disagreements between the two reviewers were resolved by consultation with a third independent reviewer.

Literature traceability: Systematic literature traceability was performed for all included secondary studies focusing on depression-associated gut microbiota. The original references supporting microbiota-depression conclusions cited in these secondary studies were targeted for layered source tracing to identify their original data sources and analytical foundations. First, all cited original studies referenced in secondary literature were retrieved and prioritized to verify whether they provided primary experimental data. All newly identified original studies obtained through traceability were re-screened strictly following the established PRISMA workflow and predefined inclusion and exclusion criteria.

Data extraction: A standardized unified data extraction form was adopted to extract valid information from all eligible studies. For all qualified original literature, we systematically extracted gut microbial detection indicators, mainly including the relative abundance of gut microbiota and bacterial genera or species with statistically significant intergroup differences. After completing literature traceability and incorporating newly identified original studies, duplicate records were removed. All newly included primary studies were extracted following the identical standardized criteria. Consistent extraction caliber and complete data entries were ensured throughout the process to generate standardized and comprehensive basic data for subsequent microbial differential analysis and evidence quality evaluation.

### 2.4 Literature quality evaluation and evidence grading

This study adopts the classification quality evaluation and unified evidence grading mode, strictly following the provided rating system (Table 2). According to the GRADE system scoring standard, comprehensive study design, quality evaluation and result consistency, all evidence is uniformly divided into 4 grades: A (High), B (Moderate), C (Low), and D (Very low). Grade A (High): The reliability of results is extremely high, and the true effect is close to the estimated value. Grade B (Moderate): The reliability is moderate, and the true effect may be close to the estimated value. Grade C (Low): Limited confidence, true effects may differ significantly from estimates. Grade D (Very low): Little confidence, conclusions unreliable.

**TABLE 2:** Literature evidence grading table.

Grade		Description	RCT	Cross-sectional study	Case reports
A	High	Very confident that the true effect size is close to the effect estimate	All factors present (bias in the randomization process, bias in deviation from the intended intervention, bias in missing outcome data, bias in outcome measurement, bias in selective reporting of results)	All factors present (sample inclusion criteria, standard criteria, validity of exposure measurement, measurement of outcomes, control of confounding factors, description of study participants, appropriateness of statistical methods)	
B	Moderate	Moderate confidence in the effect estimate: the true value is likely close to the estimate, but there remains a possibility that the two are significantly different	One factor missing	One factor missing	
C	Low	Limited confidence in the effect estimate: the true value may be significantly different from the estimate	Two factors missing	Two factors missing	
D	Very Low	Almost no confidence in the effect estimate: the true value is likely significantly different from the estimate	Three factors missing	Three factors missing	D

The RCT adopted Cochrane ROB 2.0 Bias Risk Assessment Tool (Sterne et al., 2019), and the quality evaluation of cross-sectional studies adopted JBI (Joanna Briggs Institute) evaluation criteria for cross-sectional studies (Barker et al., 2026). Although individual case reports were also included, because they were observational, uncontrolled and non-standardized retrospective descriptive studies, the results of only a few cases reported were easily interfered by individual differences, underlying diseases and other factors, so the individual case reports were directly rated as D.

## 3 Results

### 3.1 Literature search screening and literature traceability process

**FIGURE 1:**
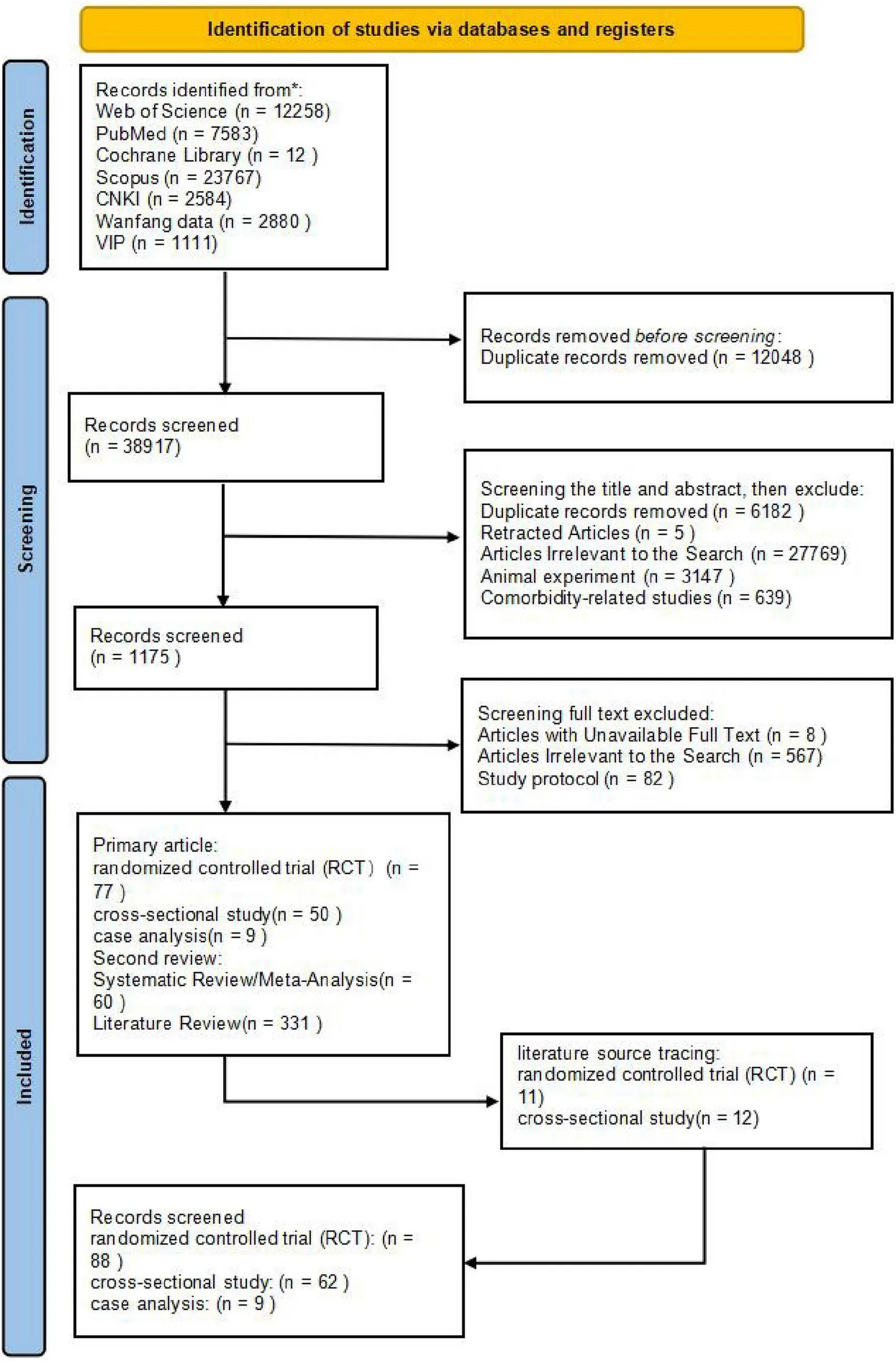
Flow chart of literature search screening and literature traceability.

**FIGURE 2:**
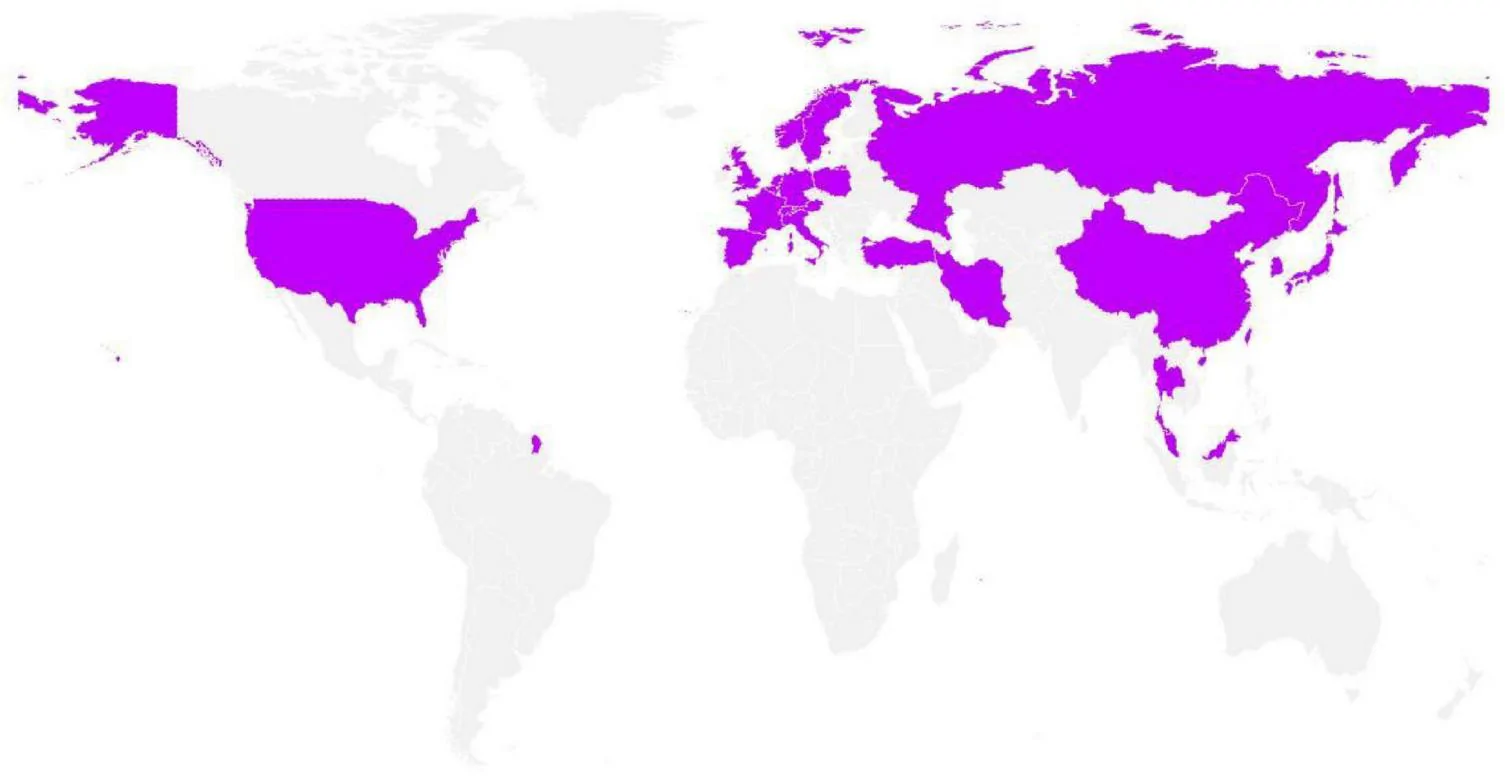
Regional distribution of countries. Purple areas indicate countries of included studies; grey areas denote countries/regions without relevant included literature.

Literature screening was performed in accordance with the PRISMA guidelines. A total of 50,965 records were initially retrieved from public databases, among which 12,048 duplicate articles were removed using EndNote 2025. After preliminary screening by titles and abstracts, 6,182 additional manually identified duplicates, 27,769 irrelevant records, 3,147 animal experimental studies, 5 retracted articles, and 639 studies involving comorbid diseases were further excluded, leaving 1,175 articles for full-text screening. During the full-text review, 567 irrelevant studies, 82 study protocols, and 8 articles without accessible full texts were eliminated, while no duplicate or data-unavailable records were excluded at this stage. Ultimately, 127 original studies were retained after full-text screening. Furthermore, a total of 391 secondary studies were traced back to their primary sources, and 32 additional eligible original articles were supplemented after deduplication. In total, 159 qualified studies were included in the present analysis (Figure 1), covering 25,310 patients with depression across 21 countries in Europe, North America, and East Asia (Figure 2).

### 3.2 Taxonomic distribution, abundance shifts and evidence level assessment of domain bacteria gut microbiota in patients with depression

#### 3.2.1 Taxonomic distribution of domain bacteria gut microbiota in patients with depression

**FIGURE 3:**
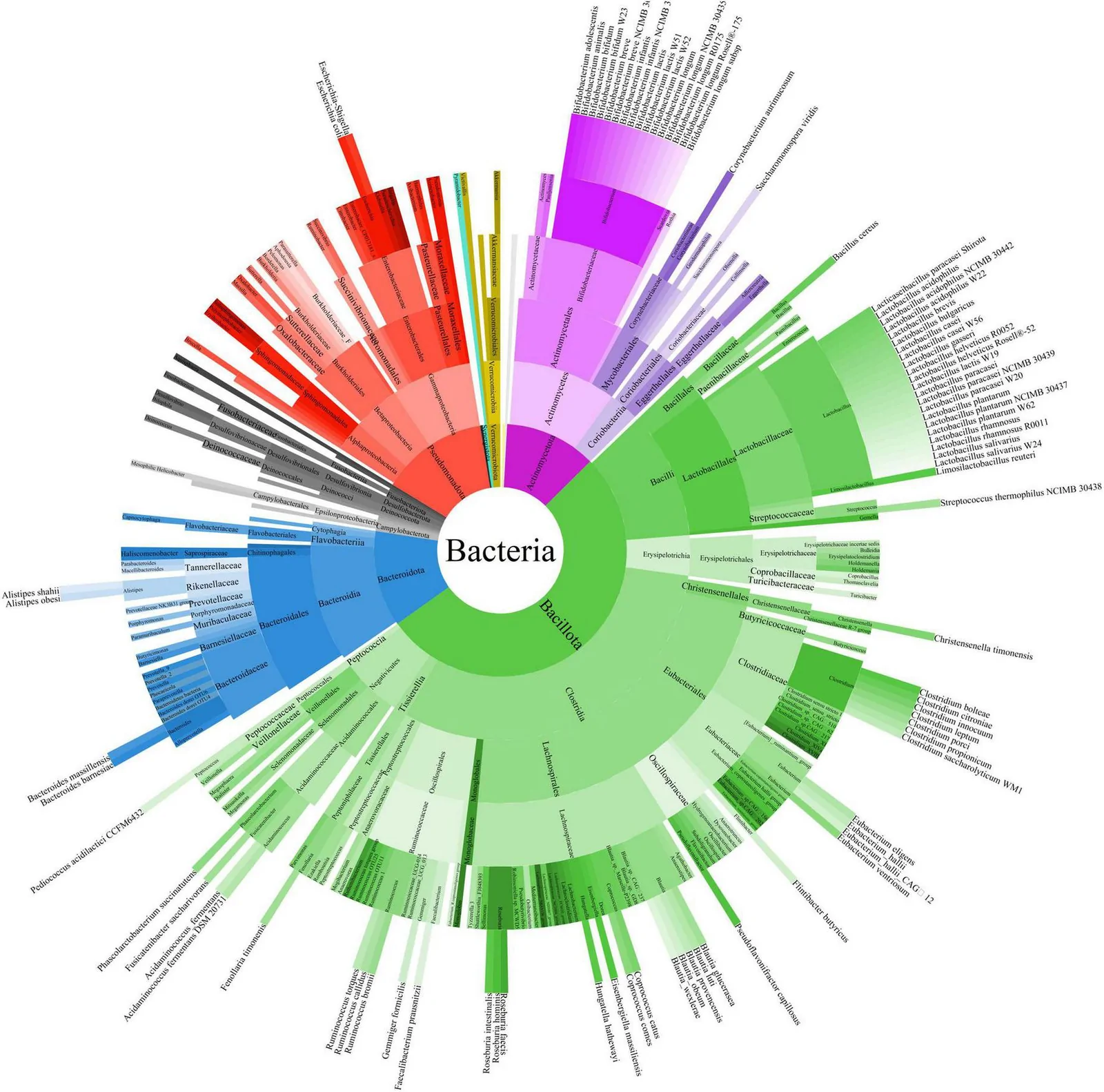
Hierarchical classification diagram of gut microbiota from Domain Bacteria in patients with depression. The concentric rings display seven taxonomic ranks from the inner to outer circle: domain, phylum, class, order, family, genus, and species. Sector area is positively correlated with the occurrence frequency of microbial taxa, and different colored sectors represent distinct phyla. Green sectors denote Bacillota, blue sectors denote *Bacteroidota*, purple sectors denote *Actinomycetota*, and red sectors denote *Pseudomonadota*.

A circular classification tree (sunburst diagram) was applied in this study to visualize the taxonomic structure of gut microbiota in patients with depression, systematically illustrating community profiles of bacteria and fungi from kingdom down to the species level. Within the included dataset, the bacterial community under depressive conditions comprised 14 phyla, 19 classes, 33 orders, 72 families, 152 genera, and 87 species (Figure 3).

The phyla identified were: *Bacillota, Actinomycetota, Acidobacteriota*, *Bacteroidota*, *Campylobacteria, Cyanobacteria*, *Deferribacteria*, *Fusarium*, *Mycoplasmatota, Patescibacteria*, *Pseudomonas*, *Spirochaetota, Synergistota*, *Verrucomicrobiota*. As shown in Figure 3, the green sector is *Bacillota*, the blue sector is *Bacteroidota*, the purple sector is *Actinomycetota*, the red sector is *Pseudomonas*. *Bacillota* accounts for about 1/2 of the gut microbiota of depressed patients, and *Bacillota* is a closely related phylum.

The main classes included: *Bacilli*, *Clostridia*, *Erysipelotrichia*, *Negativicutes*, *Selenomonadales*, *Clostridia*, *Bacteroidia*, *Coriobacteriia*, *Gammaproteobacteria*, *Bacteroidia*, *Flavobacteriia*, *Cytophagia*, *Sphingobacteriia*, *Coriobacteriia*, *Actinomycetes*, *Gammaproteobacteria*, *Epsilonproteobacteria* and others belong to *Clostridia* of *Bacillota*. *Bacilli* predominate in the green area of the pie chart; *Bacteroidia* are predominant within *Bacteroidota*. *Actinomycetes* and *Coriobacteriia* are the main classes of *Actinomycetota*, and *Gammaproteobacteria* have the highest abundance in *Pseudomonadota.*

The main orders included: *Clostridiales*, *Lactobacillales*, *Bifidobacteriales*, *Bacteroidales*, *Coriobacteriales*, *Enterobacterales*, *Christensenellales*, *Eggerthellales*, *Erysipelotrichales*, *Selenomonadales*, *Veillonellales*, *Burkholderiales*, *Campylobacterales*, *Desulfovibrionales* and others. Among these, *Clostridiales* is the dominant order within *Clostridia*; *Lactobacillales* are commonly found in *Bacilli*; *Bacteroidales* is a prevalent order within *Bacteroidota*; and *Coriobacteriales* and *Enterobacterales* are the dominant orders within *Coriobacteriia* and *Gammaproteobacteria*, respectively.

The main bacterial families included: *Lachnospiraceae*, *Ruminococcaceae*, *Lactobacillaceae*, *Bifidobacteriaceae*, *Bacteroidaceae*, *Muribaculaceae*, *Prevotellaceae*, *Coriobacteriaceae*, *Eggerthellaceae*, *Enterobacteriaceae*, *Clostridiaceae*, *Eubacteriaceae*, *Veillonellaceae*, *Acidaminococcaceae*, *Christensenellaceae*, *Streptococcaceae* and others. Among them, *Lachnospiraceae* and *Ruminococcaceae* are the two dominant butyrate-producing core families within the order *Clostridiales*; *Lactobacillaceae* and *Streptococcaceae* are the predominant families in the order *Lactobacillales*; *Bacteroidaceae* and *Prevotellaceae* are the major families within the order *Bacteroidales*; *Coriobacteriaceae* and *Eggerthellaceae* are the core families of the order *Coriobacteriales*; and *Enterobacteriaceae* is the dominant family within the order *Enterobacterales*.

The genus level comprises a large number of taxa, totaling 135, with the major key genera including: *Bifidobacterium*, *Lactobacillus*, *Blautia*, *Clostridium*, *Roseburia*, *Faecalibacterium*, *Eubacterium*, *Bacteroides*, *Ruminococcus*, *Streptococcus*, *Enterococcus*, *Christensenella*, *Collinsella*, *Eggerthella*, *Acidaminococcus*, *Desulfovibrio*, *Escherichia*, *Akkermansia*, *Prevotella*, *Alistipes*, and others. Among them, *Bifidobacterium* and *Lactobacillus* are dominant probiotic genera in the gut, primarily clustered within the order *Lactobacillales*; *Blautia*, *Roseburia*, *Faecalibacterium*, and *Eubacterium* are the predominant butyrate-producing genera within *Clostridiales*; *Bacteroides*, *Prevotella*, and *Alistipes* represent core polysaccharide-degrading genera in *Bacteroidales*; *Christensenella* is the hallmark genus of the family *Christensenellaceae*; and *Enterobacteriaceae* is predominantly represented by Escherichia.

At the species level, the primary differentially abundant gut bacteria linked to depression are: *Bifidobacterium longum, Bifidobacterium breve, Bifidobacterium infantis, Bifidobacterium bifidum, Lactobacillus acidophilus, Lactiplantibacillus plantarum, Lacticaseibacillus rhamnosus, Lacticaseibacillus paracasei, Lactobacillus gasseri, Lactobacillus helveticus, Levilactobacillus brevis, Phocaeicola dorei, Phocaeicola barnesiae* and others. At the species level, *Bifidobacterium* is a highly abundant core dominant species.

Subspecies and strains are identified through genomic classification, commonly used to elucidate disease mechanisms. Causality is then verified via *in vitro* cell and animal experiments. The study identified subspecies such as *Bifidobacterium infantis* and *Bifidobacterium lactis*, as well as *Bifidobacterium longum-related strains* including *Bifidobacterium longum NCIMB 30435*, *Bifidobacterium longum R0175*, and *Bifidobacterium longum Rosell^®^-175, along with *Lactiplantibacillus plantarum*-related strains such as *Lactiplantibacillus plantarum NCIMB 30437* and *Lactiplantibacillus plantarum W62*. *Bacteroidota* and *Bifidobacteria* are central to subspecies and strain research.*

Meanwhile, this study identified a few microbial sequences with standardized Latin names: metagenome-binned genomes *(bin_31, bin_32, bin_55, UBA1819)*, 16S rRNA sequence clusters assigned to provisional groups (*Family_XIII_AD3011_group*, *SMB53*), and uncultivated tentative species units from the GTDB database (*LLKB_g_PAC001047_s*, *PAC001103_g_PAC001103_s*).

#### 3.2.2 Abundance shifts and evidence level assessment of gut microbiota in patients with depression

##### 3.2.2.1 Bacillota

*Bacillota* represents the most abundant phylum in the gut microbiome of individuals with depression. Results indicate that the abundance of most butyrate-producing commensal bacteria is downregulated, while a small subset of pro-inflammatory pathogenic clades is upregulated—with these associations supported by high-level evidence (Grades A and B). Specifically, bar charts for *Butyricicoccus, Faecalibacterium*, and genera within the family *Lachnospiraceae* (e.g., *Dorea*, *Lachnospiraceae_FCS020_group*, *Pseudobutyrivibrio, Roseburia*, *Tyzzerella3*) extend to the left, supported by robust Grade A and B evidence, reflecting a pronounced trend of reduced abundance. In contrast, bar charts for *Acidaminococcus*, *Enterococcus, Oscillospira*, and other genera extend to the right, also backed by high-grade evidence (Grades A and B), indicating a significant increase in abundance. The genus *Eubacterium* exhibits a distinct bidirectional abundance pattern: certain subclades and species (including *Eubacterium halliigroup*, *Eubacterium eligens*, *Eubacterium ventriosum*, *Eubacterium_hallii_CAG:12*, and *Eubacterium_hallii*) show strong support for decreased abundance, whereas clades such as Eubacterium_g23, *Eubacterium*_sp. *CAG:156*, and *Eubacterium*_sp. *CAG:202* demonstrate evidence of increased abundance. *Lactobacillus* exhibits an overall decrease in the gut microbiota of individuals with depression, though a small number of studies report an upward trend. Bar plots for multiple Lactobacillus species and unclassified operational taxonomic units (OTUs), including *Lacticaseibacillus paracasei Shirota*, *Lactobacillus acidophilus*, *Levilactobacillus brevis*, *Lactobacillus bulgaricus*, *Lacticaseibacillus casei*, and *Lacticaseibacillus casei W56*, extend predominantly to the left, with most supported by Grade A or B evidence. This indicates a clear trend of reduced abundance and high-quality evidence, identifying these as core butyrate-producing commensal taxa within the phylum *Bacillota* that are significantly depleted in depression-associated microbial dysbiosis. Fragments with low evidence levels (Grades C and D) are only sporadically distributed across individual taxonomic units and do not alter the core trend of overall downregulation for this genus.

**FIGURE 4:**
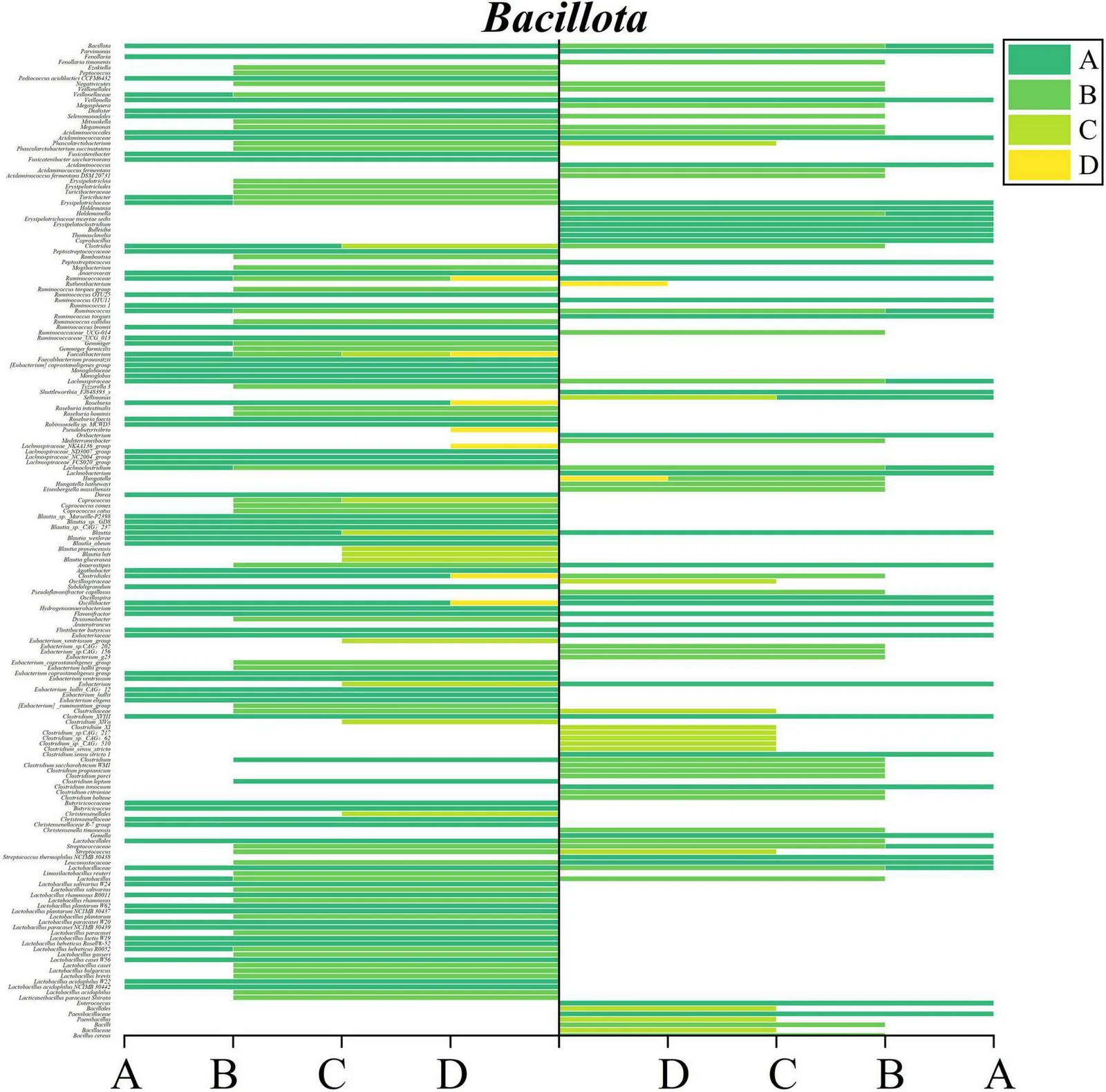
Changes in the abundance of bacterial phylum *Bacillota* and evidence grading in patients with major depressive disorder. The stacked bar plot shows abundance alterations and evidence grading for bacterial phylum *Bacillota* in patients with major depressive disorder. Taxonomic names are displayed vertically, while evidence confidence grades (Grade D to Grade A) are displayed horizontally. Stacked bars extending leftward toward Grade A represent decreased relative abundance of target taxa, while stacked bars extending rightward toward Grade A represent increased relative abundance of target taxa.

*Clostridium*shows complex bidirectional changes in abundance in the gut microbiota of patients with depression, with marked differences in evidential support. certain clostridiumclades and species, such as *Clostridium porci*, *Clostridium propionicum*, *Clostridium sensu stricto*, and *Clostridium* sp. *CAG:510*—exhibit a significant increase in depressed individuals, while other clades/species (e.g., *Clostridium leptum* and Clostridium XIVa) show a marked overall decrease in abundance. In addition, multiple genera within the phylum *Bacillota* display bidirectional shifts in microbial abundance. Beyond the abovementioned taxa, these include *Phascolarctobacterium*, Clostridium XVIII, *Anaerostipes*, *Blautia*, *Coprococcus*, *Lachnoclostridium*, *Oscillibacter*, *Flavonifractor*, *Ruminococcus*, *Megamonas*, *Streptococcus*, and *Veillonella*, all supported by high-level evidence (Figure 4).

##### 3.2.2.2 Other bacterial phyla

Although *Bacillota* constitutes the dominant phylum in the gut microbiota of individuals with depression, other phyla including *Actinomycetota*, *Bacteroidota*, *Pseudomonadota*, *Verrucomicrobiota*, and *Thermodesulfobacteriota* also exhibit trends of dysbiosis. The results are as follows:

Within the phylum *Actinomycetota*, certain genera and species (including *Actinomyces*, *Scardovia*, *Rothia*, and *Corynebacterium aurimucosum*) exhibit a significant increase in microbial abundance, supported by Grade A or B evidence. While the genus *Bifidobacterium* displays opposing bidirectional trends in abundance, multiple species and unclassified operational taxonomic units (OTUs) within this genus (such as *Bifidobacterium animalis*, *Bifidobacterium bifidum*, *Bifidobacterium bifidumW23*, *Bifidobacterium breve*, *Bifidobacterium breve NCIMB 30441*, *Bifidobacterium infantis*, *Bifidobacterium lactis*, *Bifidobacterium lactisW51*, *Bifidobacterium longum*, and *Bifidobacterium longumR0175*) show a significant reduction in abundance. The bidirectional shifts in *Collinsella* abundance are supported by high grade evidence.

Within the phylum *Bacteroidota*, a bidirectional trend in microbial abundance was also observed: genera and species represented by *Barnesiella*, *Phocaeicola*, *Alloprevotella*, and *Alistipes shahii* exhibited decreased abundance, whereas *Paraprevotella*, *Porphyromonas*, and *Alistipes obesi* showed increased gut microbial abundance. Notably, the genus *Bacteroides*, especially its distinct species, displayed a divergent bidirectional pattern, with both increased and decreased abundance supported by Grade A and Grade B evidence. Similarly, *Prevotella* lacked a consistent directional trend, with conflicting Grade A and Grade B evidence for its abundance changes, making it one of the genera with heterogeneous responses in the gut microbiota of individuals with depression. The same heterogeneous pattern was also observed in *Alistipes* and *Parabacteroides*.

Within the phylum *Pseudomonadota*, the abundance of taxa represented by *Sutterella* and *Pseudomonas* was strongly supported to be decreased, while the abundance of pathogenic taxa, primarily *Escherichia*, *Escherichia-Shigella*, and *Escherichia coli*, is strongly supported to be increased. *Enterobacter* has high-grade evidence supporting bidirectional changes in abundance. For Parasutterella, only high-grade evidence supports increased abundance, whereas the evidence for a decreasing trend is limited to Grade C/D, which warrants further investigation.

Beyond the aforementioned phyla, a small number of other bacterial phyla also exhibited trends of dysbiosis, with significant variations in microbial abundance. The bar charts for *Campylobacteraceae*, *Fusobacteriaceae*, and *Terrimicrobiaceae* shifted to the right, supported by Grade A/B evidence, indicating strong support for increased abundance. In contrast, *Akkermansia*, a genus within the family *Akkermansiaceae*, showed conflicting bidirectional evidence regarding its abundance changes.

**FIGURE 5:**
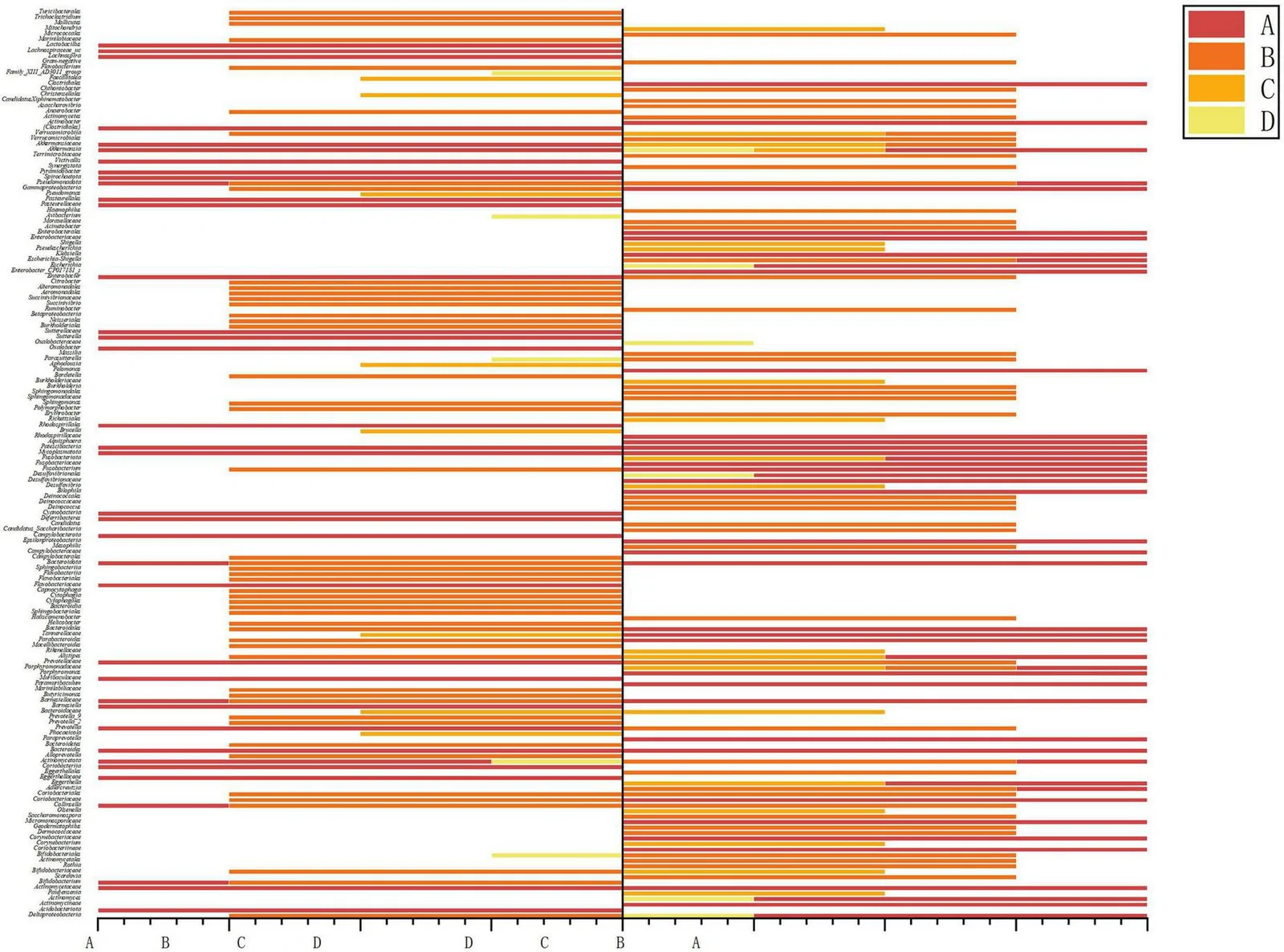
Changes in gut microbial abundance and evidence grading of other phyla within Bacteria in patients with major depressive disorder. The stacked-bar plot illustrating abundance alterations and evidence grading for other phyla within Bacteria in patients with major depressive disorder. Taxonomic names are displayed vertically, while evidence confidence grades (Grade D to Grade A) are displayed horizontally. Stacked bars extending leftward toward Grade A indicate decreased relative abundance of target taxa, whereas bars extending rightward toward Grade A indicate increased relative abundance of target taxa.

Most differentially abundant microbial taxa are supported by Grade A or B evidence, indicating high credibility of their abundance change results. However, a small subset of taxa still exhibits conflicting bidirectional evidence. In contrast to the extensive genus and species level research data available for *Bacillota*, analyses of the other aforementioned phyla are mostly restricted to the family-level classification, with limited detailed evidence at the genus or species level (Figure 5).

#### 3.2.3 Hierarchical structure grading of gut microbiota within bacteria in patients with major depressive disorder

##### 3.2.3.1 Bacillota

**FIGURE 6:**
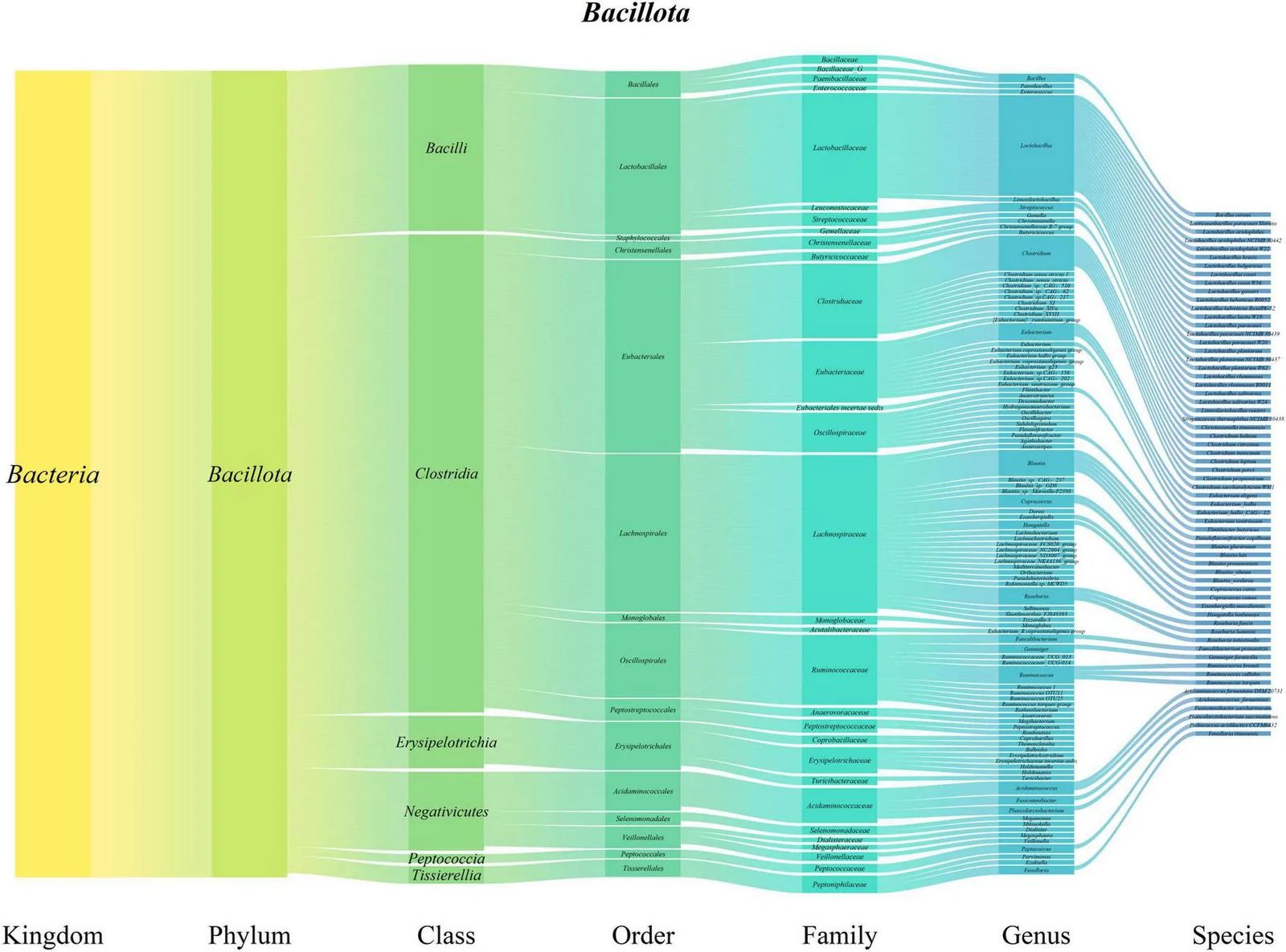
Sankey diagram of *Bacillota* within bacteria in patients with major depressive disorder. Presents a taxonomic hierarchical structure diagram for *Bacillota* within bacteria in patients with major depressive disorder. The Sankey flows run from left to right across seven taxonomic ranks, from phylum to species. Flow widths quantify the occurrence frequency of microbial taxa. *Clostridia* and *Bacilli* represent the dominant classes within *Bacillota*, with markedly wider flows than other classes, and they further give rise to multiple core orders and families at successive taxonomic levels.

To intuitively compare the hierarchical structural differences within the phylum *Bacillota*, a seven-level taxonomic sankey diagram was constructed (domain-phylum-class-order-family-genus-species). The width of flow lines quantifies the total relative abundance of taxonomic units in each group, while microbial evidence grades (Figure 4) were integrated to systematically characterize the *Bacillota* phylum (Figure 6). Overall, the gut *Bacillota* community in individuals with depression is dominated by two core classes: *Clostridia* and *Bacilli*. Notably, the *Clostridia* lineage exhibits the highest number of sub-branches and the largest flow width, indicating it is the most species-diverse class within *Bacillota*, followed by *Bacilli*. The orders *Lachnospirales*, *Lactobacillales*, *Eubacteriales*, and *Oscillospirales* constitute the core taxonomic orders of the depressive gut microbiome, extending downward to dominant families including *Lachnospiraceae*, *Lactobacillaceae*, *Clostridiaceae*, and *Ruminococcaceae.* While the abundance of some gut microbes within *Bacillota* increases at the genus level (e.g.), *Lachnobacterium*, *Mediterraneibacter*, *Oribacterium*), most taxa show a decreasing trend at the species level. These include species under the genus Lactobacillus (e.g., *Lactobacillus acidophilus*, *Lactobacillus acidophilus W22*, *Levilactobacillus brevis*, *Lactobacillus bulgaricus*, *Lacticaseibacillus casei W56*) and species under the genus Blautia (e.g.), *Blautia luti*, *Blautia provencensis*, *Blautia obeum*, *Blautia wexlerae*). Given the strong supporting evidence for these abundance changes, these species could be regarded as putative candidate taxa for probiotic-assisted intervention in major depressive disorder (Supplementary Table 1).

##### 3.2.3.2 Other bacterial phyla

**FIGURE 7:**
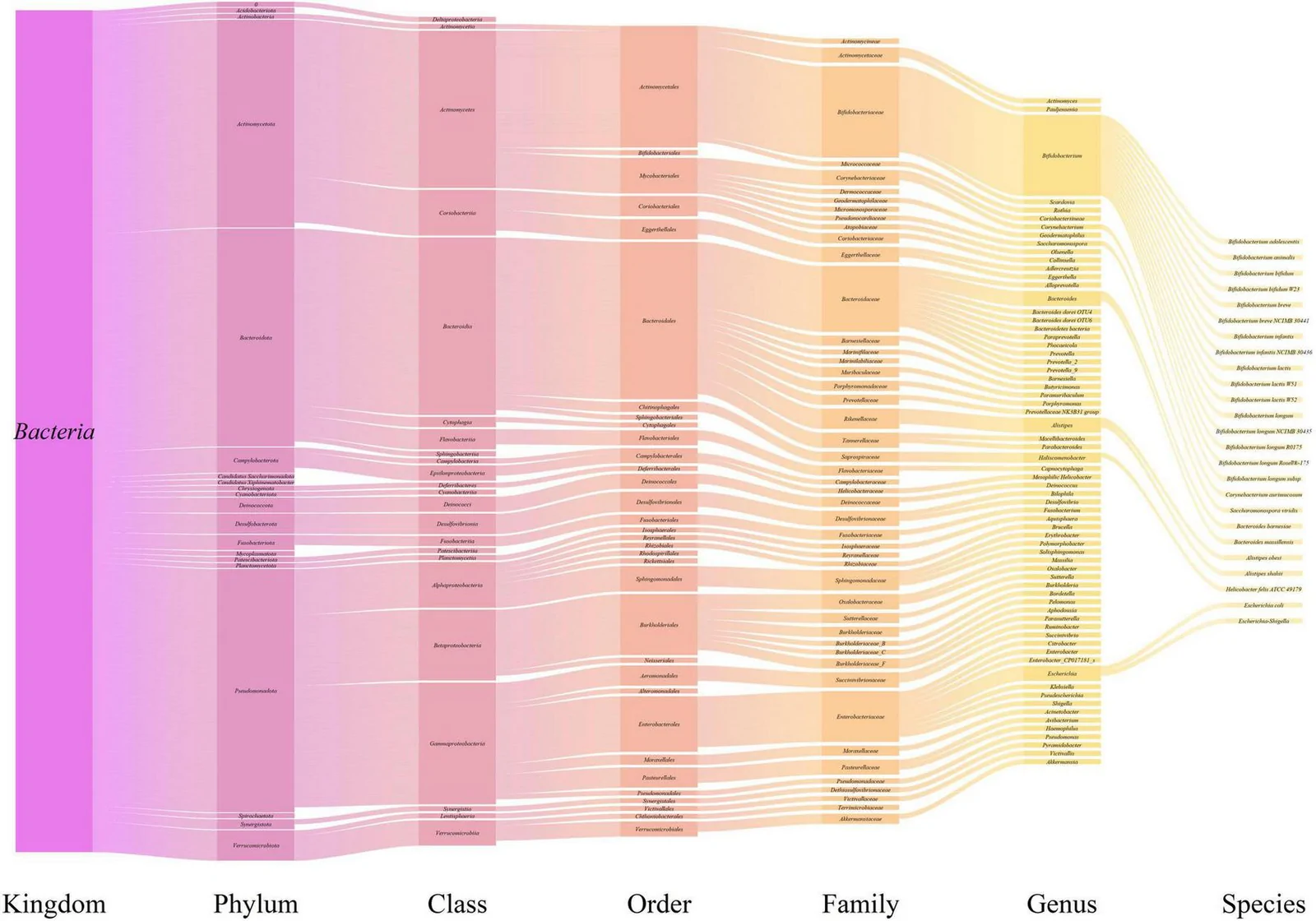
Sankey diagram of other phyla within bacteria in patients with major depressive disorder. Presents the taxonomic hierarchical structure of other phyla within bacteria in patients with major depressive disorder. The Sankey flows extend from left to right across seven taxonomic ranks, from phylum to species. Flow widths quantify the occurrence frequency of microbial taxa. *Actinobacteriota*, *Pseudomonadota* and *Bacteroidota* represent the dominant phyla among bacteria in patients with major depressive disorder, with markedly wider flows than other minor phyla.

Combining Figures 5, 7, the overall results demonstrate that, in addition to *Bacillota*, the gut microbiota of individuals with depression is primarily dominated by three core phyla: *Actinobacteriota*, *Pseudomonadota*, and *Bacteroidota*. *Actinobacteriota*is predominantly composed of the class *Actinomycetes*, with the order *Bifidobacteriales* serving as its core taxonomic unit. The families *Bifidobacteriaceae* and genus *Bifidobacterium* exhibit the widest flow widths, representing the absolute dominant groups within this phylum. *Proteobacteria* is dominated by the class *Gammaproteobacteria*, with the orders *Enterobacterales* and Pseudomonadales forming prominent branches. In contrast, *Verrucomicrobiota*, *Spirochaetota*, *Deferribacterota*, and *Fusobacteriota* display narrow flow widths and are classified as rare gut microbial taxa. Collectively, these observations indicate a phylum-level structural imbalance in the gut microbiota of individuals with depression. *Bacteroidota* consists solely of the class *Bacteroidia* and order *Bacteroidales*, which extend downward to dominant families including *Bacteroidaceae*, *Prevotellaceae*, and *Flavobacteriaceae*. Notably, *Bifidobacterium* and its subordinate species (e.g., *Bifidobacterium adolescentis*, *Bifidobacterium animalis*, *Bifidobacterium bifidum*, *Bifidobacterium breve*), species of *Bacteroides* (e.g., *Bacteroides massiliensis*, *Bacteroides barnesiae*), and the genera *Prevotella_2* and *Prevotella_9* all exhibit a predominantly decreasing trend in abundance. Supported by robust evidence, these could offer potential perspectives for future research and development of microecological preparations (Supplementary Table 2).

### 3.3 Distribution characteristics, abundance alterations, and evidence grading of gut microorganisms within fungi in patients with major depressive disorder

#### 3.3.1 Distribution characteristics of gut microorganisms within fungi in patients with major depressive disorder

**FIGURE 8:**
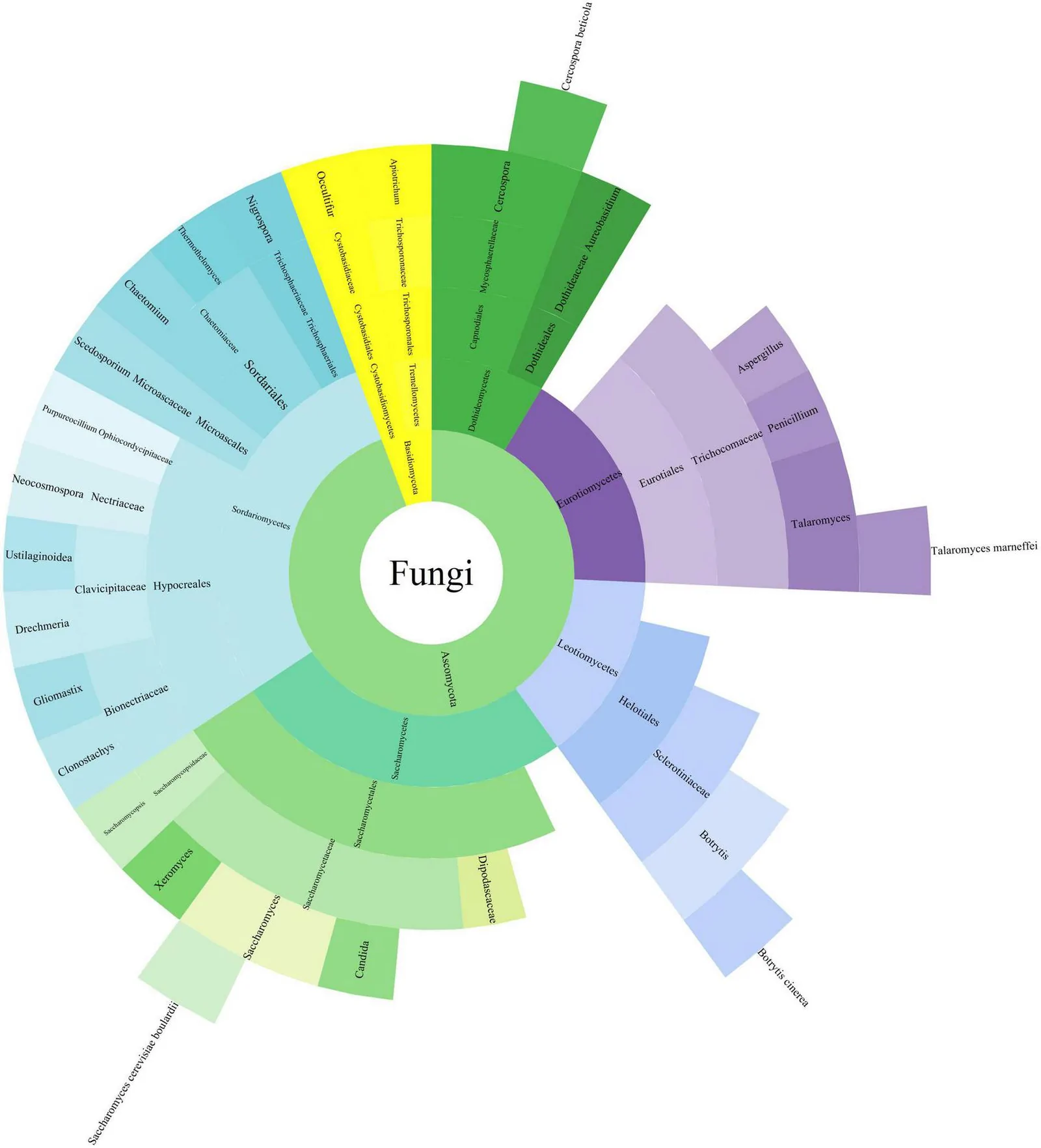
Hierarchical taxonomic plot of gut microorganisms within fungi in patients with major depressive disorder. Displays seven taxonomic ranks (kingdom, phylum, class, order, family, genus, species) arranged concentrically from inner to outer rings. The sector area is positively correlated with the occurrence frequency of microbial taxa, and sectors with distinct color blocks are used to differentiate major phyla.

Fungal communities are affiliated with the kingdom Fungi (Figure 8). Within the gut fungal community, the phylum *Ascomycota* constitutes the dominant group. At the class level, *Saccharomycetes*, *Eurotiomycetes*, and *Leotiomycetes* are the predominant classes, among which *Saccharomycetes* is the most abundant and represents the absolute dominant class, with representative genera including *Candida*, *Saccharomyces*, *Xeromyces*, and *Clavispora*. This is followed by the family *Trichocomaceae* (within *Eurotiomycetes*), represented by the genera *Aspergillus*, *Penicillium*, and *Talaromyces*, as well as the genera *Botrytis* and *Sclerotinia* from the class *Leotiomycetes*.

The results indicate that *Candida* is the most core enriched fungal genus in the gut microbiota of depression patients, and could be considered as a potential diagnostic feature for depressive disorders in future studies. However, overall fungal community diversity remains low, and the causal relationship between fungal dysbiosis and depression still needs to be further validated.

#### 3.3.2 Alterations in fungal gut microbial abundance and evidence grading in patients with major depressive disorder

**FIGURE 9:**
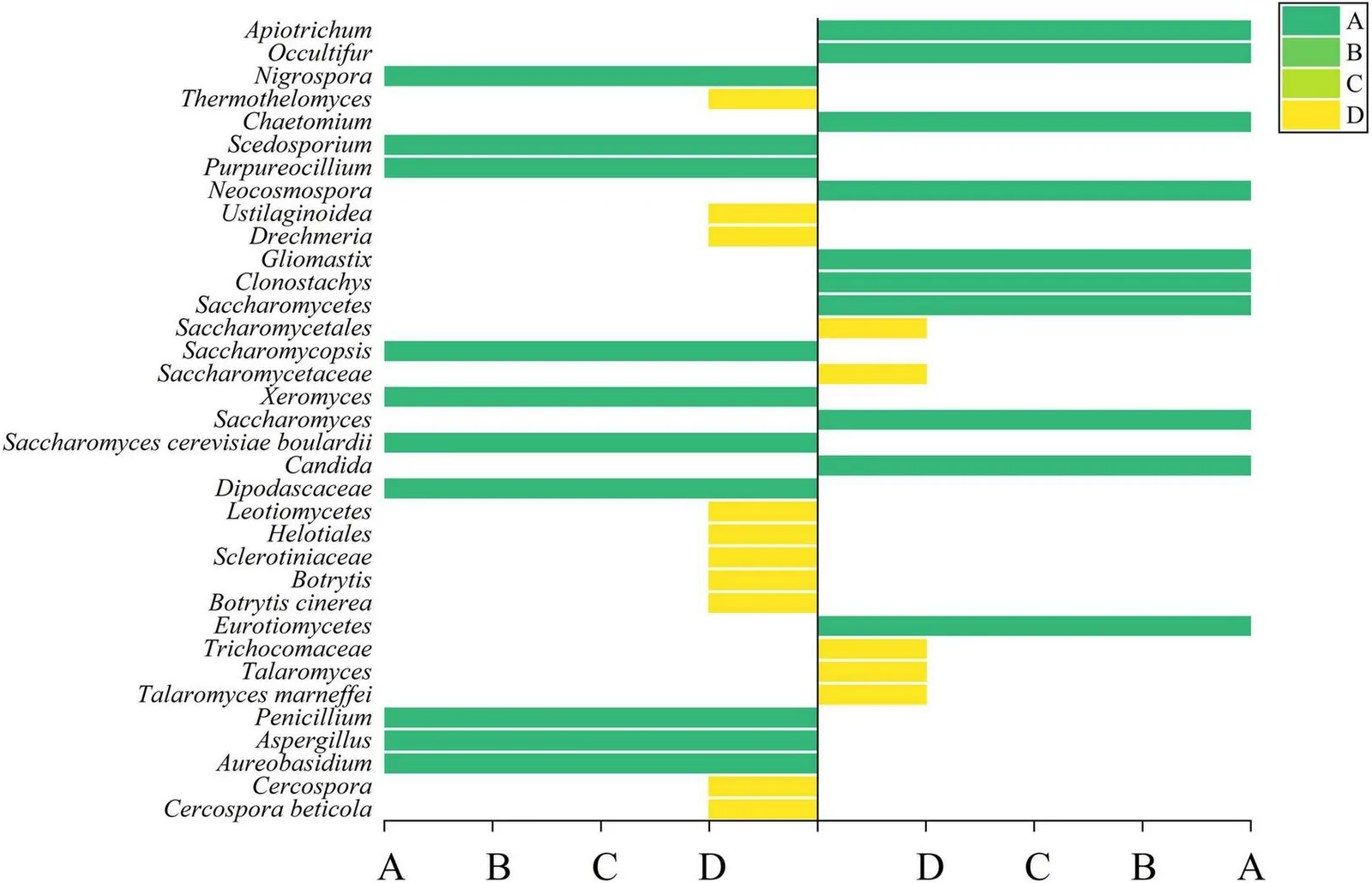
Changes in fungal gut microbial abundance and evidence grading in patients with major depressive disorder. The stacked-bar plot illustrating abundance alterations and evidence grading for gut microorganisms within fungi in patients with major depressive disorder. Taxonomic names are displayed vertically, while evidence confidence grades (Grade D to Grade A) are displayed horizontally. Stacked bars extending leftward toward Grade A indicate decreased relative abundance of target taxa, whereas bars extending rightward toward Grade A indicate increased relative abundance of target taxa.

*Ascomycota* represents the dominant fungal phylum in depression-related research, exhibiting bidirectional patterns of microbial abundance across distinct taxonomic levels, with marked discrepancies in the strength of supporting evidence. At the genus level, *Saccharomyces cerevisiae boulardii* is supported by high-grade (Grade A and B) evidence for a significant reduction in abundance, while the majority of studies are confined to the family and genus taxonomic ranks. Specifically, at the genus level, the decreasing trends of *Aureobasidium*, *Aspergillus*, *Penicillium*, *Saccharomyces cerevisiae boulardii*, *Xeromyces*, *Saccharomycopsis*, *Purpureocillium*, *Scedosporium*, and *Nigrospora* are collectively supported by Grade A and B evidence. In contrast, *Candida*, *Saccharomyces*, *Clonostachys*, *Gliomastix*, *Neocosmospora*, *Chaetomium*, *Occultifur*, and *Apiotrichum* show rightward shifts in bar charts, with concurrent Grade A and B evidence indicating increased abundance in depression. Notably, for a small subset of taxa, evidence supporting either increased or decreased abundance is limited to Grade C or D, warranting further investigation (Figure 9).

#### 3.3.3 Hierarchical structure grading of gut microbiota within fungi in patients with major depressive disorder

**FIGURE 10:**
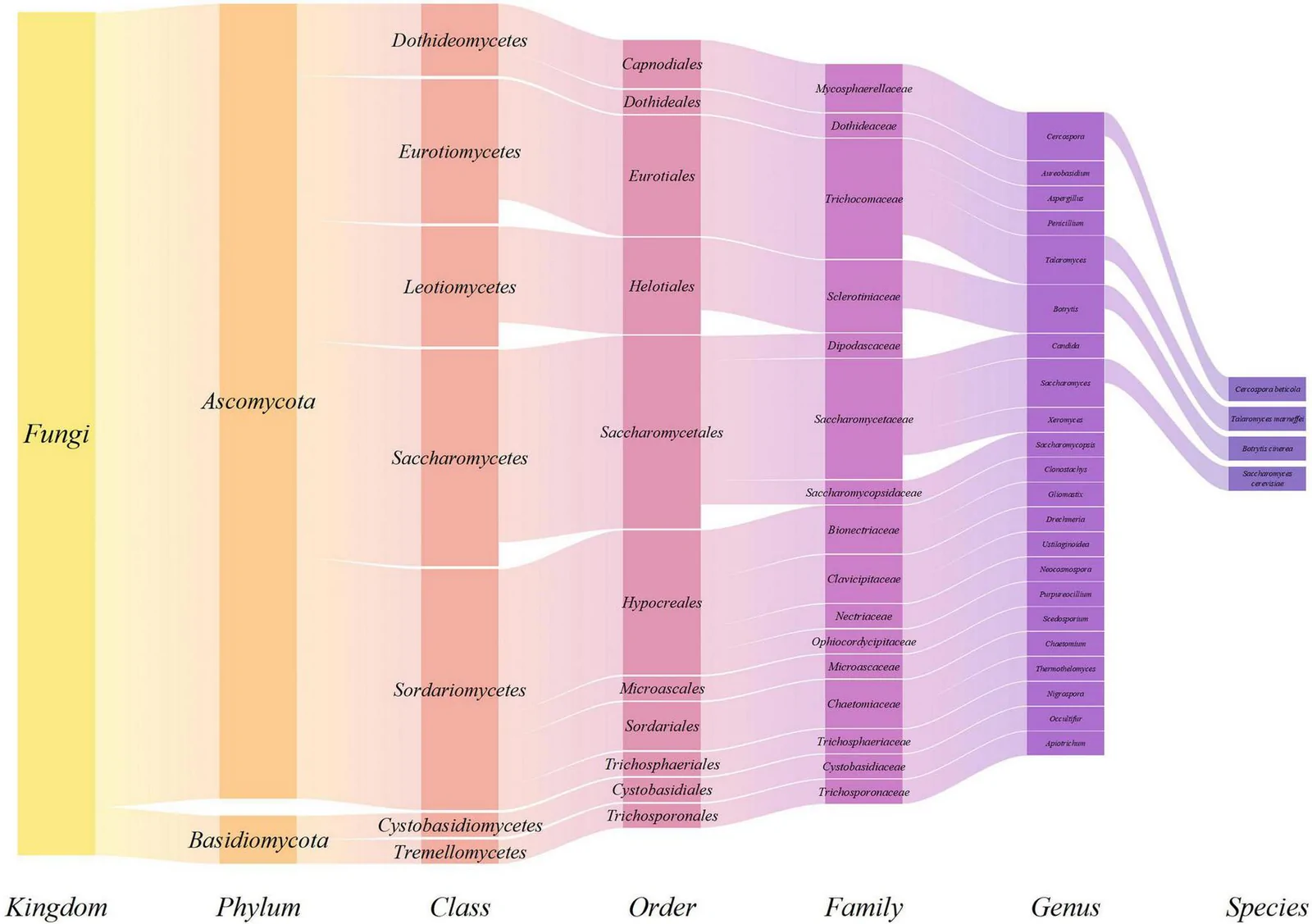
Sankey diagram of gut microorganisms within fungi in patients with major depressive disorder. Presents a taxonomic hierarchical structure diagram for fungal taxa in patients with major depressive disorder. The Sankey flows run from left to right across seven taxonomic ranks, from kingdom to species. Flow widths quantify the occurrence frequency of microbial taxa. *Ascomycota* constitutes the absolutely dominant phylum within fungi in patients with major depressive disorder, with markedly wider flows than other minor phyla, and it further gives rise to multiple core orders and families at successive taxonomic levels.

Integrating data from Figures 9, 10, the fungal community is dominated by *Ascomycota*, whereas *Basidiomycota* exhibits significantly lower species diversity. Within *Ascomycota*, seven core classes are identified, among which *Saccharomycetes*, *Eurotiomycetes*, and *Leotiomycetes* display the closest associations with the human microbiome. Notably, while multiple genera within the yeast class (*Saccharomycetes*) show a decreasing trend, the genus *Saccharomyces* remains the only taxon across the fungal kingdom that includes medicinal probiotic strains. Specifically, *Saccharomyces cerevisiae var. boulardii is* supported by robust clinical evidence for its application as an oral probiotic. The remaining genera are categorized into three functional groups: Opportunistic human pathogens: e.g., *Candida* spp. and *Talaromyces marneffei*; Plant pathogens: e.g., *Cercospora* spp. and *Botrytis* spp.; Industrial fermentation strains: e.g., *Aspergillus* spp. and *Penicillium* spp., which are exclusively used in food or pharmaceutical fermentation processes. None of these latter groups are suitable for development as intestinal probiotic preparations (Supplementary Table 3).

## 4 Probiotics

Integrating data from Figures 5–10 and focusing on species-level microbial abundance changes, a total of 87 species-level taxa (encompassing both bacteria and fungi) were analyzed. Approximately 75% (66 taxa) exhibited reduced abundance, among which over 50% were supported by Grade A/B evidence. The bidirectional opposing patterns of species-level abundance changes represent a central focus of probiotic research. Notably, species within the genus *Bifidobacterium* (including *Bifidobacterium adolescentis*, *Bifidobacterium bifidum*, *Bifidobacterium bifidum W23*, *Bifidobacterium breve*, and *Bifidobacterium infantis*) showed significant reductions in abundance. Additionally, anti-inflammatory and butyrate-producing taxa such as *Lacticaseibacillus paracasei Shirota*, *Lactobacillus plantarum*, *Flintibacter butyricus*, and *Saccharomyces cerevisiae boulardii* also displayed marked decreases. In contrast, pathogenic taxa including *Escherichia-Shigella*, *Clostridium innocuum*, and *Candida* spp., exhibited significant increases in abundance. Future microbiome-targeted biotherapeutics could restore gut microbial homeostasis by enhancing the abundance of protective taxa and inhibiting the overgrowth of pathogenic or harmful microbes. This, in turn, may alleviate clinical symptoms of depression and slow disease progression.

## 5 Discussion

Based on existing literature evaluation systems, the present study conducted a data visualization analysis of depression-related gut microbiome research. We aimed to comprehensively classify and grade the evidence level of all reported differential microbial taxa, and to construct a visual knowledge map of gut dysbiosis in major depressive disorder. By synthesizing the changes in gut microbial abundance observed in clinical studies of depressed patient populations, our work provides valuable reference for clarifying the specific microbiome distribution of dysbiosis in depression, exploring the mechanisms underlying heterogeneous microbial alterations, and developing targeted microbial intervention agents. Our findings are as follows: First, gut dysbiosis in depression is characterized by a dual imbalance of both bacterial and fungal communities, although the depth of research on fungi lags considerably behind that on bacteria. The gut microbial imbalance is primarily manifested as a decline in protective taxa and enrichment of pathogenic bacteria, with the reduction of protective commensals being a predominant feature of the dysbiotic state. Second, existing studies on gut microbiota in depression have largely focused on *Lactobacillus* (within *Bacillota*), *Bifidobacterium* (within *Actinobacteria*), *Enterobacter* (within *Proteobacteria*), *Bacteroides* (within *Bacteroidota*), as well as the fungal genera *Saccharomyces* and *Candida*. Third, although most findings are consistent across studies, some taxa still show heterogeneous abundance changes, and further multidimensional experiments are needed to clarify the causal relationships underlying these variations. Finally, based on the dysbiotic features of gut microbiota in depressed patients, we discuss the potential value of probiotics as adjunctive interventions and propose rational recommendations for future directions in gut microbiota research.

### 5.1 Gut microbiota dysbiosis represents the core manifestation of intestinal microecological perturbations in depression

The gut microbiome mainly encompasses bacteria, fungi, viruses, archaea, and protozoa. Since 2009, when the O’Mahony team first demonstrated through animal experiments that stress and other stressors can disrupt microbial homeostasis and induce depressive behaviors (O’Mahony et al., 2009), Subsequent researchers have focused on the gut microbiota and depression, conducting extensive clinical and mechanistic studies. Most research indicates that the gut microbiota in individuals with depression shows significant imbalance compared to healthy individuals. The earliest clinical study was initiated by Naseribafrouei and his team in 2014 (Naseribafrouei et al., 2014). Through 16S rRNA gene sequencing, it was confirmed at the human level that depressed patients have dysbiosis and microbial imbalance; subsequently, Jiang et al., also conducted a cross-sectional study (Jiang et al., 2015), further clarifying of the microbial imbalance in depressed patients and revealing a significant decline in the genus *Faecalibacterium*. As research progresses, scientists have found that fungal dysbiosis is also one of the characteristics of gut microbiota imbalance in patients with depression (Jiang et al., 2020), mainly characterized by an increase in *Candida* and a decrease in *Penicillium*. Fungi and bacteria coexist and interact within the fungal-bacterial microenvironment (Peleg et al., 2010; MacAlpine et al., 2023). Existing studies have reported disrupted fungal-bacterial interaction networks in patients experiencing depressive episodes (Hao et al., 2023), suggesting that homeostatic disturbance of these two communities may be partially associated with the pathogenesis of major depressive disorder. Enteric viruses are abundant in the gastrointestinal tract, with comparable quantities to bacteria. Evidence has indicated perturbed bacteria-virus-metabolite crosstalk in the gastrointestinal tract of patients with bipolar depression (Kong et al., 2026) Animal-model studies (Ritz et al., 2024) have further demonstrated alterations in enteric viruses among chronically-stressed mice. Nevertheless, constrained by detection methodologies, only a limited number of studies employing gut-viral metagenomic sequencing have identified links between enteric viruses and major depressive disorder (Zhang et al., 2026; Simpson et al., 2021). Increased abundances of s_Stenotrophomonas_virus_Pokken, g_Pokkenvirus, s_Dickeya_virus_AD1, and g_Alexandravirus in the gut microbiota of depressed patients represent the core findings from current relevant investigations.

Although parasitic protozoa inhabit the human gut, reports linking them to depression remain relatively scarce, which may be attributed to advances in modern medical care and improvements in human dietary habits. Nevertheless, Toxoplasma gondii, an exogenous parasite, has been suggested to bear potential associations with the onset of depression in several studies (Ramírez-Carrillo et al., 2020). Moreover, a recent groundbreaking cohort study has for the first time detected archaea in the gut microbiota of individuals with depression (Motger-Albertí et al., 2026). The study indicates a close association between the severity of depression in female patients and M*ethanogenic archaea*. However, this study only establishes a correlation between archaea and depression, rather than a causal relationship. Future large-sample clinical studies and in-depth mechanistic research are required to elucidate the relationships between viruses, protozoa, archaea and gut microbiota in individuals with major depressive disorder.

Bacteria have remained the central focus of research on gut microbiota dysbiosis. While inconsistencies exist across studies (Zhang et al., 2026), the most consistent conclusion is the depletion of protective bacteria (e.g., short-chain fatty acid (SCFA)-producing bacteria, and butyrate-producing bacteria) triggered by inflammation (Simpson et al., 2021; Cao et al., 2025; Nikolova et al., 2021). At the genus level, our study not only confirmed the decreased abundance of well-characterized butyrate-producing protective genera (e.g., *Faecalibacterium, Butyricicoccus*, *Dialister*, *Roseburia*, Fusicatenibacter), a finding consistent with prior research—but also identified a significant downward trend in several unclassified taxa, including *Lachnospiraceae_FCS020_group*, *Lachnospiraceae_NC2004_group*, *Ruminococcaceae_UCG_013*, *Blautia_sp._GD8*, and *ChristensenellaceaeR-7 group*. At the species level, over 50% of microbial taxa exhibited a significant reduction in abundance. These taxa were predominantly clustered within: (1) the genus *Bifidobacterium* (e.g., *Bifidobacterium adolescentis*, *Bifidobacterium breve*); (2) the genus *Lactobacillus* (e.g., *Lacticaseibacillus casei*); and (3) beneficial butyrate-producing species, such as *Blautia luti*, *Blautia glucerasea*, *Faecalibacterium prausnitzii*, *Roseburia intestinalis*, and *Ruminococcus bromii*.

### 5.2 Mechanisms linking the onset of major depressive disorder to the reduction of beneficial taxa

The findings indicate that the gut microbial community structure in patients with depression is markedly imbalanced, prominently manifested as a decline in the abundance of multiple protective commensal taxa, including short-chain fatty acid-producing bacteria affiliated with *Bacillota*, certain species within the genus *Lactobacillus*, and certain species within the genus *Bifidobacterium.* The depletion of these protective gut commensals may, via gut-brain axis-related cascade pathways, trigger neuroinflammation and impairments in neuroplasticity, thereby contributing to the pathogenesis of depression.

Studies have suggested that gut microbial alterations lead to increased production of inflammation-related lipopolysaccharide (Erny et al., 2015) and elevated serum levels of pro-inflammatory cytokines, such as interleukins (IL), in patients with depression (Osimo et al., 2020), which in turn activate the vagus nerve and transmit neuroinflammatory signals to the central nervous system (Fries et al., 2023), thereby exacerbating depressive symptoms. Gut microbiota-derived short-chain fatty acids (SCFAs) play a crucial role in the gut-brain axis regulation of depression. SCFAs, particularly butyrate, can cross the blood-brain barrier and exert anti-inflammatory and neuroprotective effects (Cheng et al., 2024). Depletion of SCFA-producing protective commensals leads to insufficient SCFA production, impairs intestinal barrier integrity, and induces the occurrence of “leaky gut.” As potent inhibitors of histone deacetylases (HDACs), reduced SCFA levels resulting from the loss of protective bacteria attenuate HDAC inhibition, which in turn decreases histone acetylation, upregulates pro-inflammatory gene transcription, and impairs intestinal regulatory T-cell (Treg) immune tolerance, thereby amplifying peripheral inflammatory responses. Peripheral pro-inflammatory cytokines can enter the central nervous system through the compromised blood–brain barrier and vagal afferent pathways, subsequently inducing excessive microglial activation in the hippocampus and prefrontal cortex, and ultimately triggering central neuroinflammation.

For example, *Faecalibacterium*, a strictly obligate anaerobic Gram-positive bacillus (Shakir Shakir et al., 2026), is one of the most important butyrate-producing bacteria in the human gut (Barandouzi et al., 2020). It is widely distributed in the gut microbiota, accounting for more than 5% of the total microbial community (Chang et al., 2022). A reduction in its abundance leads to insufficient SCFA production in the gut, which impairs the integrity of intestinal epithelial tight junctions and increases intestinal permeability. This, in turn, promotes the translocation of endotoxins into the bloodstream via the “leaky gut” mechanism, thereby triggering systemic low-grade inflammation (Papacocea and Ciobanu, 2026; Luu et al., 2026) and enhancing the production of inflammation-related lipopolysaccharide (Erny et al., 2015). Subsequently, the vagus nerve is activated, transmitting neuroinflammatory signals to the central nervous system (Fries et al., 2023). Concurrently, the inflammatory state upregulates indoleamine 2,3-dioxygenase (IDO) activity, driving tryptophan metabolism away from the 5-HT synthesis pathway toward the neurotoxic kynurenine pathway. At the same time, it weakens Treg-mediated intestinal immune tolerance and overactivates the HPA axis, resulting in persistently elevated cortisol levels, disruption of the blood-brain barrier, and ultimately neuroinflammation. These cascading events eventually induce or exacerbate depression-related emotional and behavioral abnormalities (Niemela et al., 2024; Hu et al., 2023).

The genus *Lactobacillusis* recognized as a group of protective beneficial commensals in the gut (Shah et al., 2024; Gao et al., 2022). Although the abundance changes of this genus at the taxonomic level exhibit heterogeneity, studies at the species level indicate that multiple species within this genus show a decreasing trend in abundance changes among depressed patients. Moreover, different *Lactobacillus* strains participate in the regulation of depression through distinct mechanisms, including modulation of intestinal barrier function, anti-inflammatory effects, and neuro-metabolic regulation (Li et al., 2026; Joung et al., 2025; Kaur et al., 2023). Recent studies have suggested that *Lactobacillus rhamnosus* and its related strains, *Lactobacillus reuteri* and *Lactobacillus rhamnosus GG* (Feng et al., 2025; Wang et al., 2024), can enhance intestinal barrier integrity by upregulating tight junction proteins such as Claudin-4 and Occludin, while concurrently ameliorating depressive and anxiety-like behaviors. Another study reported that *Lacticaseibacillus casei* intervention ameliorated stress-induced depressive-like behaviors in rats and restored the expression of monoamine neurotransmitters (including dopamine, norepinephrine, and serotonin) in the brain by activating the BDNF-TrkB signaling pathway and modulating gut microbiota composition (Gu et al., 2020). Gamma-aminobutyric acid (GABA), the primary inhibitory neurotransmitter in the central nervous system, is critically involved in emotional regulation and stress responses. In depressed patients, decreased GABA levels and dysfunction of GABA_a_ receptors are frequently observed in emotion-related brain regions. Exogenous GABA administration has been shown to improve depressive symptoms, and among the gut microbiota, *Levilactobacillus brevisis* recognized as a potential GABA-producing protective taxon (Paul et al., 2026). Furthermore, animal studies have indicated that GABA produced via *Levilactobacillus brevis* fermentation mediates antidepressant-like effects in depressive model mice, accompanied by inhibition of monoamine oxidase activity and reduction of stress hormones and neuroinflammation (Kim et al., 2024).

Multiple species within the genus *Bifidobacterium*, as one of the beneficial commensals in the gut, have been found to be depleted in association with depressive behaviors. Among them, *Bifidobacterium adolescentis*, a representative species, shows significantly reduced abundance in the gut microbiota of depressed patients (Mueller et al., 2006; Chen et al., 2018). Depletion of this bacterium leads to insufficient secretion of SCFAs, such as acetate and propionate, disrupts the intestinal epithelial barrier, causes lipopolysaccharide (LPS) leakage (Aizawa et al., 2016), and induces systemic and central chronic neuroinflammation. Concurrently, it upregulates indoleamine 2,3-dioxygenase (IDO) activity, shifting tryptophan metabolism toward the neurotoxic kynurenine pathway, and impairs vagal gut-brain signaling (Arbabi et al., 2024; Chung et al., 2019). Conversely, *Bifidobacterium* supplementation has been shown to produce acetylcholine, GABA, and serotonin, thereby alleviating depressive symptoms (O’Neill et al., 2023).

**FIGURE 11:**
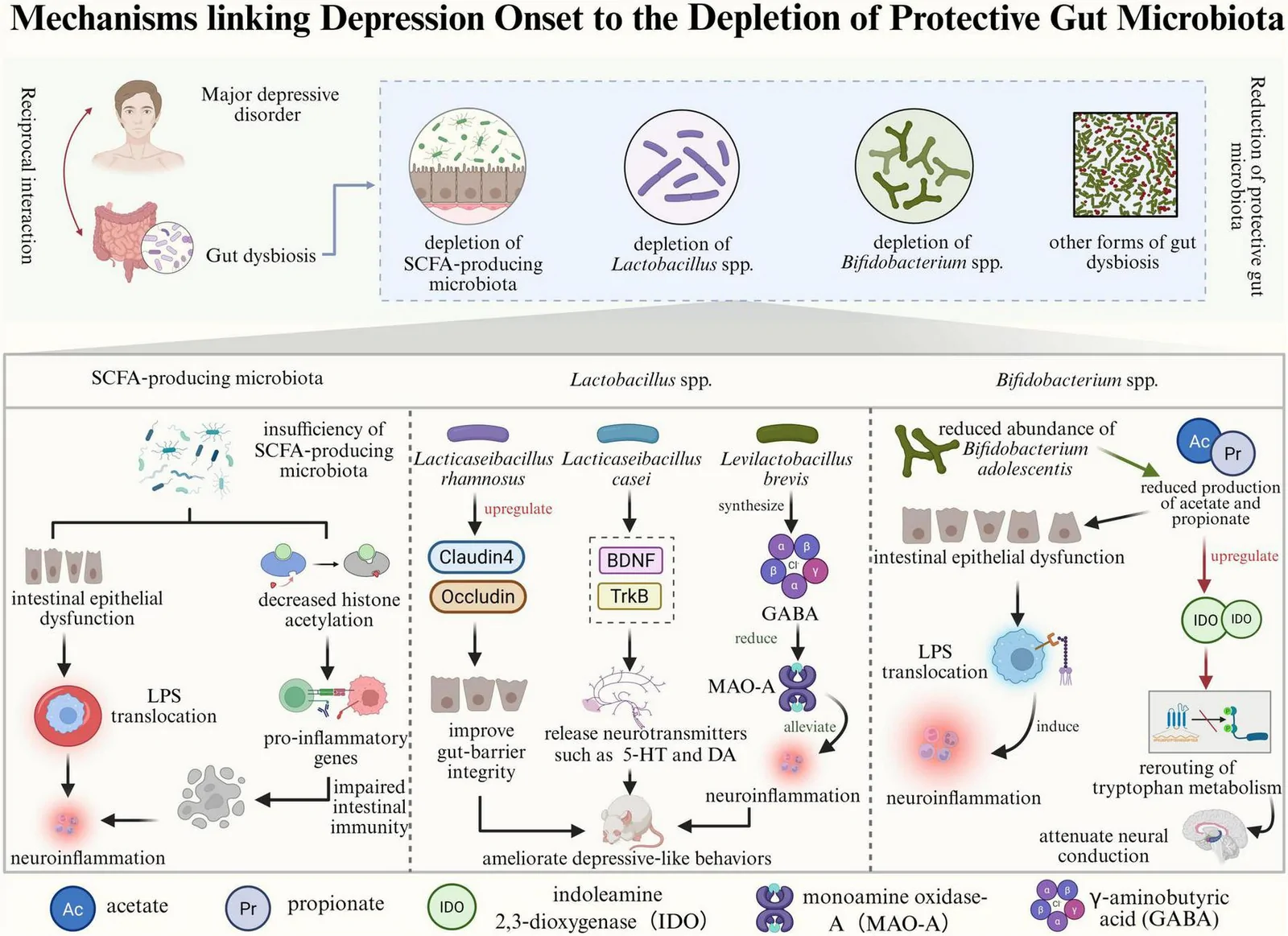
Mechanisms underlying the onset of depression and the decline in protective commensal abundance. The gut microbiota in patients with depression is markedly imbalanced, with a predominant feature being the reduced abundance of protective commensals. This reduction primarily involves decreased levels of short-chain fatty acid (SCFA)-producing bacteria, as well as certain species within the genera *Lactobacillus* and *Bifidobacterium*. The decline in protective commensals mainly disrupts the intestinal barrier, allowing inflammatory factors such as LPS to enter the bloodstream and induce both peripheral and central inflammation, thereby contributing to the development of depression. Conversely, supplementation with specific bacterial strains can alleviate depressive symptoms by activating relevant molecular pathways, reducing inflammation, and promoting neurotransmitter release. Created in BioRender. Xu, C. (2026) [https://BioRender.com/ujqqe9b].

In summary, depression and the decline in abundance of protective commensals are interrelated and interact with each other. The underlying mechanisms primarily involve reduced production of SCFs (Cheng et al., 2022), induction of inflammatory responses (Shakir Shakir et al., 2026), immune dysregulation, and dysregulation of neurotransmitters (Chang et al., 2022; Figure 11).

### 5.3 Genus level shifts in the abundance of taxon specific gut microbiota

Although most studies show consistent results at the species level, there is conflicting evidence regarding changes in microbial abundance at the genus level (Barandouzi et al., 2020; Papacocea and Ciobanu, 2026; Luu et al., 2026). For example, genera such as *Bifidobacterium*, *Bacteroides*, *Clostridium*, and *Enterobacter* exhibit divergent findings. The heterogeneity of research findings may be attributable to variations in sex, dietary patterns, medication status, geographical regions, depression severity, and age (Niemela et al., 2024; Hu et al., 2023; Mueller et al., 2006). Some studies suggest that *Actinobacteria* phylum and *Bacteroidota* class show increased and decreased trends, respectively, in female and male patients (Chen et al., 2018), potentially serving as gender-specific biomarkers for diagnosing depression. Among them, *Bifidobacterium* is a specific genus within the *Actinobacteria* phylum. While its abundance consistently decreases at the species level, studies on the *Bifidobacterium* genus in depressed individuals report contradictory outcomes, some showing reduced abundance, others elevated. For instance, comparing fecal microbiota between depressed patients and healthy controls, several studies found significantly lower levels of *Bifidobacterium* in depressed individuals (Aizawa et al., 2016; Arbabi et al., 2024). However, Chung et al. (2019) reported a significant increase in *Bifidobacterium* abundance among depressed subjects. Knudsen et al. (2021) further confirmed this bidirectional pattern through systematic review, suggesting that differences in dietary habits, microbiome detection methods, and analytical approaches might explain these inconsistencies, though no causal relationship was established. Similarly, changes in *Bacteroides* abundance among depressed patients also display opposing trends closely linked to gender differences. A gender-stratified clinical study revealed that *Bacteroides* levels were significantly reduced in male patients but relatively higher in females (Chen et al., 2018). Dietary structure is another influencing factor: O’Neill et al. (2023) observed lower Bacteroides abundance in depressed patients, yet those following a low-FODMAP diet showed enriched Bacteroides levels. Additionally, Borgiani et al. (2025) indicated that antidepressant use can affect microbial abundance and gut microbiota diversity. Du et al. (2026) using metagenomic sequencing, compared different responders to the same medication and found that their gut microbial compositions exhibited abundance differences in opposite directions. Regional differences also contribute to bidirectional shifts in microbial abundance. Gao et al. (2023) compared populations from eastern and western regions to identify microbial changes in depression, finding inconsistent patterns in genera such as *Clostridium* and *Enterobacter*, particularly in East Asian studies, especially those conducted in China. Age difference is another important influencing factor, and the distribution and abundance of gut microbiota in patients with depression show significant differences across different age groups (Chen J. J. et al., 2020). Zhao et al. (2024) reported higher *Bacteroides* abundance in younger patients, while older patients exhibited the opposite trend. The bidirectional changes in microbial abundance are influenced by multiple factors, with gender, age, and regional differences being the main contributors. Future primary research should employ more rigorous subgroup analyses or standardized, controlled conditions to minimize confounding effects and reduce conflicting results.

### 5.4 Future perspectives

Pharmacological therapy (Vas et al., 2023), psychological interventions (Mirchandaney et al., 2022), and physical stimulation therapies (Hsieh, 2023) are currently the mainstream approaches for treating depression. With advancing research on the microbiome, gut microbial agents, as an adjunctive therapeutic measure for depression, have received preliminary support from several clinical studies (Tian et al., 2022). Biotherapeutics targeting the gut microbiota are gradually gaining recognition as a novel adjunctive intervention strategy. At present, most biotherapeutic development focuses on exogenous supplementation of beneficial symbiotic bacteria, such as *Lactobacillus* and *Bifidobacterium*. However, depressive patients often exhibit dysbiosis characterized by increased levels of pro-inflammatory and pathogenic microbes. Moreover, most existing microbiome studies remain limited to genus-level species analysis, while investigations into strain- or species-specific mechanisms remain relatively scarce. Given these research gaps, this article suggests that future basic and translational research could be further advanced along two key dimensions.

#### 5.4.1 Development of microbial agents as adjunctive interventions for depression.

Depression, a mental disorder with extremely high disability risk, affects over 300 million people worldwide (“Global, regional, and national burden of 12 mental disorders in 204 countries and territories, 1990–2019: a systematic analysis for the Global Burden of Disease Study 2019” GBD 2019 Mental Disorders Collaborators, 2022). Although oral medications remain the first-line clinical treatment, deeper research into the microbiome has revealed that long-term use of these drugs can alter gut microbial activity and bioavailability, thereby reducing therapeutic efficacy (Weersma et al., 2020). Probiotic interventions show promising potential (Liu et al., 2023). Multiple preliminary clinical studies have indicated that interventions with microbial agents can concurrently regulate both the mood and gut microecology of patients with depression, suggesting a potential association between gut microbiota and the pathogenesis of depression (Paul et al., 2026; Socała et al., 2021; Asad et al., 2025). Yet current research still faces several key challenges. First, different strains exhibit specific modes of action. Second, significant differences exist among depressed patients in terms of age, gender, and dietary habits. Additionally, research targeting pro-inflammatory or pathogenic bacteria remains relatively limited. Based on the above research status, it might be considered that future work could attempt to establish a relatively standardized strain screening and evaluation system, to explore individualized intervention regimens that take into account age and sex differences, and to investigate approaches for bidirectional modulation of the gut microbiota, potentially with a view to gradually restoring the disturbed intestinal microecology in patients with depression.

#### 5.4.2 Microbiome research: deepening from genus to species

Research findings from a number of studies suggest that changes in gut microbiota abundance at the species level appear to be relatively consistent across most investigations, whereas results at the genus level have shown discrepancies, with some findings even being contradictory. These opposing results may limit our in-depth understanding of the gut microbiota in depression. Although it might be possible to clarify genus-level outcomes by controlling for external confounding factors, conducting species-level analyses could still be of importance for elucidating the microbial mechanisms underlying depressive disorder. Current species-level research primarily focuses on taxa within genera such as *Lactobacillus*, *Bifidobacterium*, *Blautia*, and *Clostridium*. Our findings reveal significant differences in abundance changes among genera within *Lachnospiraceae* and *Ruminococcaceae*, yet species-level investigations remain relatively scarce. Given that these two groups are commonly found in depressed patients, conducting species-level studies on them represents a meaningful innovation. To advance microbiome research from genus to species level, the following approaches can be adopted: (1) Upgrading detection technologies, replacing 16S rRNA amplicon sequencing with high-throughput shotgun metagenomic sequencing to enable more precise identification at the species or even strain level. Combined with metabolomics, this approach allows for the analysis of associations between specific microbial metabolites and depressive phenotypes. (2) Functional validation experiments, using germ-free mouse colonization models to verify the causal roles of specific bacterial species in depressive phenotypes.

### 5.5 Limitation

However, our study is not without limitations. First, due to the wide geographical span of the included studies, differences in dietary habits and BMI among participant populations, together with confounding factors such as sequencing protocols, types of antidepressants, and combination regimens that are difficult to fully control, the variations across studies in controlling for these confounders, including diet, medication, and geographic location—may introduce bias into the pooled results of gut microbiota analyses. Second, given the paucity of reporting on non-bacterial microorganisms (e.g., archaea, viruses) in primary studies, our evidence grading focuses predominantly on bacteria, with only preliminary insights provided for other microbial groups such as fungi. Third, although the present study employed the Cochrane ROB 2.0 and JBI checklists for quality adjustment, this review was not prospectively registered, and methodological reporting was insufficient in some of the included studies; therefore, the conclusions should be interpreted with caution. Fourth, due to incomplete taxonomic annotations in some primary studies, where only genus- or family-level data were reported, low-resolution taxonomic units were merged during data collation, potentially resulting in the loss of species-specific information. Finally, our analysis was restricted to differentially abundant taxa and did not investigate differences in gut microbiota α-diversity indices or β-diversity (microbial community structure) between depressed patients and healthy controls. Although most studies have demonstrated significantly lower α-diversity in depressed individuals compared to healthy controls, alongside distinct β-diversity separation, residual heterogeneity across studies warrants further investigation to elucidate the underlying drivers of these conflicting findings.

## 6 Conclusion

This study systematically synthesized 159 eligible studies on the “depression-gut microbiota” axis, and the findings revealed that microbial abundance alterations in depression primarily converge on the bacterial phylum *Bacillota* and the fungal phylum *Ascomycota*. The overall signature of gut dysbiosis is characterized by the enrichment of pro-inflammatory and pathogenic taxa, accompanied by the depletion of beneficial symbiotic microbes such as butyrate-producing bacteria. Notably, contradictory results exist regarding genus-level microbial abundance changes, which are closely associated with variables including gender, age, dietary patterns, and detection/analytical methodologies across the included studies. Additionally, leveraging an evidence grading framework, this review not only identified microbial abundance trends supported by high-quality evidence but also uncovered inconsistencies in species-level research. Advancing the development of targeted microbial formulations and deepening research on underexplored microbial taxa will contribute to enhancing the clinical efficacy of microbiota-based adjuvant therapies for depression.

## Footnotes

1
https://www.ncbi.nlm.nih.gov/

2
https://gtdb.ecogenomic.org/

3
https://www.prisma-statement.org/
