# Supplementary material for: Characteristics of gut microbiota dysbiosis in patients with depression: a visual analysis of knowledge maps
Source: Front Microbiol. 2026 Sep 7;17:1937281. doi: 10.3389/fmicb.2026.1937281 (PMC13591385; doi:10.3389/fmicb.2026.1937281)
Supplement: Supplementary file 1 [file Table_1.docx]

| **Taxonomic level** | **Taxon** | **Rank** | **Gut microbiota abundance** | **Proposed function** |
| --- | --- | --- | --- | --- |
| Bacteria/Bacillota | Bacillaceae | Family | Bidirectional | Carbohydrate and protein catabolism, nutrient digestion, antimicrobial metabolite production |
| Bacteria/Bacillota | Erysipelotrichaceae | Family | Bidirectional | Participate in lipid and cholesterol metabolism, intestinal energy harvesting, and regulate mucosal immune homeostasis. |
| Bacteria/Bacillota | Lactobacillaceae | Family | Bidirectional | Carbohydrate fermentation, lactic acid production, gut barrier maintenance and mucosal immune regulation |
| Bacteria/Bacillota | Leuconostocaceae (Lactobacillaceae) | Family | Bidirectional | Carbohydrate heterofermentation, SCFA production, pathogen inhibition and immune modulation |
| Bacteria/Bacillota | Paenibacillaceae | Family | Bidirectional | Complex polysaccharide degradation, antimicrobial metabolite synthesis and immunomodulation |
| Bacteria/Bacillota | Streptococcaceae | Family | Bidirectional | Carbohydrate fermentation, lactic acid production; strain-dependent immune activation |
| Bacteria/Bacillota | Butyricicoccaceae | Family | Bidirectional | Butyrate biosynthesis, intestinal anti-inflammation and gut barrier protection |
| Bacteria/Bacillota | Christensenellaceae | Family | Bidirectional | Lipid and energy metabolism regulation, anti-inflammation and metabolic homeostasis |
| Bacteria/Bacillota | Clostridiaceae | Family | Bidirectional | Carbohydrate fermentation, SCFA/bile acid biotransformation; strain-specific pathogenic potential |
| Bacteria/Bacillota | Eubacteriaceae | Family | Bidirectional | Butyrate production, dietary fiber degradation and mucosal immune homeostasis |
| Bacteria/Bacillota | Lachnospiraceae | Family | Bidirectional | Dietary fiber degradation, SCFA synthesis, secondary bile acid transformation and immune regulation |
| Bacteria/Bacillota | Monoglobaceae | Family | Bidirectional | Pectin degradation, glycan fermentation and cross feeding with commensal microbes |
| Bacteria/Bacillota | Oscillospiraceae | Family | Bidirectional | Butyrate production, carbohydrate fermentation and anti inflammatory regulation |
| Bacteria/Bacillota | Peptostreptococcaceae | Family | Bidirectional | Amino acid fermentation, indole metabolite synthesis; strain dependent pro-inflammatory effects |
| Bacteria/Bacillota | Ruminococcaceae | Family | Bidirectional | Resistant starch/fiber degradation, SCFA production, gut microbial cross feeding |
| Bacteria/Bacillota | Turicibacteraceae | Family | Bidirectional | Bile acid biotransformation, lipid metabolism modulation and immune interaction |
| Bacteria/Bacillota | Acidaminococcaceae | Family | Bidirectional | Amino acid fermentation, propionate synthesis and gut metabolic regulation |
| Bacteria/Bacillota | Veillonellaceae | Family | Bidirectional | Lactate utilization, propionate production and short-chain fatty acid metabolism |
| Bacteria/Bacillota | [Eubacterium] _ruminantium_group | Genus | Decrease | Dietary fiber fermentation, butyrate biosynthesis and mucosal immune regulation |
| Bacteria/Bacillota | Agathobacter | Genus | Decrease | Butyrate production, anti-inflammation and intestinal barrier maintenance |
| Bacteria/Bacillota | Anaerovorax | Genus | Decrease | Amino acid fermentation, organic acid metabolism and cross feeding |
| Bacteria/Bacillota | Blautia_sp._CAG：237 | Genus | Decrease | Carbohydrate fermentation, SCFA synthesis and bile acid biotransformation |
| Bacteria/Bacillota | Blautia_sp._GD8 | Genus | Decrease | Carbohydrate fermentation, SCFA synthesis and bile acid biotransformation |
| Bacteria/Bacillota | Blautia_sp._Marseille-P2398 | Genus | Decrease | Carbohydrate fermentation, SCFA synthesis and bile acid biotransformation |
| Bacteria/Bacillota | Butyricicoccus | Genus | Decrease | Butyrate biosynthesis, anti-inflammation and gut barrier protection |
| Bacteria/Bacillota | Christensenellaceae R-7 group | Genus | Decrease | Lipid and energy metabolism modulation, anti inflammation |
| Bacteria/Bacillota | Dialister | Genus | Decrease | Amino acid fermentation, propionate synthesis and immune modulation |
| Bacteria/Bacillota | Dorea | Genus | Decrease | Carbohydrate fermentation, SCFA production and cholesterol metabolism |
| Bacteria/Bacillota | Dysosmobacter | Genus | Decrease | Butyrate synthesis, dietary polysaccharide degradation |
| Bacteria/Bacillota | Eubacterium coprostanoligenes group | Genus | Decrease | Cholesterol biotransformation, lipid metabolism regulation |
| Bacteria/Bacillota | Eubacterium hallii group | Genus | Decrease | Butyrate production, lactate utilization and metabolic homeostasis |
| Bacteria/Bacillota | Eubacterium_coprostanoligenes_group | Genus | Decrease | Cholesterol biotransformation, lipid metabolism regulation |
| Bacteria/Bacillota | Eubacterium_R coprostanoligenes group | Genus | Decrease | Cholesterol biotransformation, lipid metabolism regulation |
| Bacteria/Bacillota | Eubacterium_ventriosum_group | Genus | Decrease | Protein and amino acid fermentation, SCFA formation |
| Bacteria/Bacillota | Ezakiella | Genus | Decrease | Amino acid fermentation, proteolytic metabolism |
| Bacteria/Bacillota | Faecalibacterium | Genus | Decrease | Butyrate biosynthesis, strong anti-inflammation and gut homeostasis |
| Bacteria/Bacillota | Fenollaria | Genus | Decrease | Carbohydrate fermentation, SCFA productio |
| Bacteria/Bacillota | Fusicatenibacter | Genus | Decrease | Butyrate production, anti inflammatory immune regulation |
| Bacteria/Bacillota | Gemmiger | Genus | Decrease | Carbohydrate fermentation, butyrate synthesis |
| Bacteria/Bacillota | Hydrogenoanaerobacterium | Genus | Decrease | Anaerobic carbohydrate fermentation, hydrogen metabolism |
| Bacteria/Bacillota | Lachnospiraceae_FCS020_group | Genus | Decrease | Dietary fiber degradation, SCFA synthesis and bile acid transformation |
| Bacteria/Bacillota | Lachnospiraceae_NC2004_group | Genus | Decrease | Dietary fiber degradation, SCFA synthesis and bile acid transformation |
| Bacteria/Bacillota | Lachnospiraceae_ND3007_group | Genus | Decrease | Dietary fiber degradation, SCFA synthesis and bile acid transformation |
| Bacteria/Bacillota | Lachnospiraceae_NK4A136_group | Genus | Decrease | Dietary fiber degradation, SCFA synthesis and bile acid transformation |
| Bacteria/Bacillota | Mitsuokella | Genus | Decrease | Carbohydrate heterofermentation, organic acid production |
| Bacteria/Bacillota | Mogibacterium | Genus | Decrease | Proteolytic fermentation, amino acid metabolism |
| Bacteria/Bacillota | Monoglobus | Genus | Decrease | Pectin degradation, glycan fermentation and microbial cross feeding |
| Bacteria/Bacillota | Clostridium_XlVa | Genus | Decrease | Carbohydrate fermentation, SCFA biosynthesis; strain specific effects |
| Bacteria/Bacillota | Peptococcus | Genus | Decrease | Proteolysis, amino acid fermentation and indole metabolite formation |
| Bacteria/Bacillota | Pseudobutyrivibrio | Genus | Decrease | Hemicellulose degradation, butyrate synthesis |
| Bacteria/Bacillota | Robinsoniella sp. MCWD5 | Genus | Decrease | Carbohydrate fermentation, SCFA production |
| Bacteria/Bacillota | Romboutsia | Genus | Decrease | Carbohydrate fermentation, bile acid metabolism and immune interaction |
| Bacteria/Bacillota | Roseburia | Genus | Decrease | Butyrate biosynthesis, dietary fiber degradation and anti inflammation |
| Bacteria/Bacillota | Ruminococcaceae_UCG_013 | Genus | Decrease | Resistant starch degradation, SCFA synthesis |
| Bacteria/Bacillota | Ruminococcus 1 | Genus | Decrease | Cellulose/resistant starch degradation, SCFA production |
| Bacteria/Bacillota | Ruminococcus OTU25 | Genus | Decrease | Complex polysaccharide degradation, SCFA formation |
| Bacteria/Bacillota | Ruminococcus torques group | Genus | Decrease | Mucin glycan degradation, carbohydrate fermentation |
| Bacteria/Bacillota | Turicibacter | Genus | Decrease | Bile acid biotransformation, lipid metabolism modulation |
| Bacteria/Bacillota | Tyzzerella 3 | Genus | Decrease | Carbohydrate fermentation, SCFA synthesis |
| Bacteria/Bacillota | Subdoligranulum | Genus | Decrease | Butyrate production, fiber fermentation and anti-inflammation |
| Bacteria/Bacillota | Anaerostipes | Genus | Bidirectional | Lactate utilization, butyrate biosynthesis and intestinal anti inflammation |
| Bacteria/Bacillota | Blautia | Genus | Bidirectional | Carbohydrate fermentation, SCFA synthesis and bile acid biotransformation |
| Bacteria/Bacillota | Clostridium | Genus | Bidirectional | Carbohydrate fermentation, SCFA/bile acid transformation; strain-specific pathogenic potential |
| Bacteria/Bacillota | Clostridium_XVIII | Genus | Bidirectional | Dietary glycan fermentation, short-chain fatty acid production |
| Bacteria/Bacillota | Coprococcus | Genus | Bidirectional | Carbohydrate fermentation, butyrate synthesis and immune modulation |
| Bacteria/Bacillota | Eubacterium | Genus | Bidirectional | Dietary fiber degradation, butyrate production and mucosal immune homeostasis |
| Bacteria/Bacillota | Flavonifractor | Genus | Bidirectional | Flavonoid degradation, lipid metabolism regulation and anti-inflammation |
| Bacteria/Bacillota | Lachnoclostridium | Genus | Bidirectional | Carbohydrate fermentation, SCFA synthesis and bile acid metabolism |
| Bacteria/Bacillota | Megamonas | Genus | Bidirectional | Carbohydrate fermentation, acetate and propionate production |
| Bacteria/Bacillota | Oscillibacter | Genus | Bidirectional | Butyrate biosynthesis, anti-inflammation and gut barrier protection |
| Bacteria/Bacillota | Phascolarctobacterium | Genus | Bidirectional | Succinate utilization, propionate biosynthesis and metabolic regulation |
| Bacteria/Bacillota | Ruminococcus | Genus | Bidirectional | Resistant starch/cellulose degradation, SCFA production |
| Bacteria/Bacillota | Streptococcus | Genus | Bidirectional | Carbohydrate fermentation, lactic acid production; strain dependent immune activation |
| Bacteria/Bacillota | Veillonella | Genus | Bidirectional | Lactate consumption, propionate synthesis and SCFA metabolism |
| Bacteria/Bacillota | Lactobacillus | Genus | Bidirectional | Carbohydrate fermentation, lactic acid production, gut barrier maintenance and mucosal immune regulation |
| Bacteria/Bacillota | Acidaminococcus | Genus | Increase | Amino acid fermentation, propionate biosynthesis and gut metabolic regulation |
| Bacteria/Bacillota | Anaerotruncus | Genus | Increase | Anaerobic carbohydrate fermentation, proteolytic metabolism; opportunistic pathogenic potential |
| Bacteria/Bacillota | Bacillus | Genus | Increase | Facultative anaerobic metabolism, spore formation, enzyme and secondary metabolite biosynthesis |
| Bacteria/Bacillota | Bulleidia | Genus | Increase | Anaerobic sugar fermentation |
| Bacteria/Bacillota | Clostridium sensu stricto 1 | Genus | Increase | Anaerobic polysaccharide fermentation, SCFA biosynthesis; strain-dependent pathogenic risk |
| Bacteria/Bacillota | Clostridium_sensu_stricto | Genus | Increase | Anaerobic carbohydrate fermentationAnaerobic carbohydrate fermentation, toxin biosynthesis in pathogenic strains |
| Bacteria/Bacillota | Clostridium_sp._CAG：510 | Genus | Increase | Anaerobic carbohydrate fermentation |
| Bacteria/Bacillota | Clostridium_sp._CAG：62 | Genus | Increase | Anaerobic carbohydrate fermentation |
| Bacteria/Bacillota | Clostridium_sp.CAG：217 | Genus | Increase | Anaerobic carbohydrate fermentation |
| Bacteria/Bacillota | Clostridium_XI | Genus | Increase | rotein and amino acid fermentation |
| Bacteria/Bacillota | Coprobacillus | Genus | Increase | Carbohydrate fermentation, organic acid production and bile acid transformation |
| Bacteria/Bacillota | Enterococcus | Genus | Increase | Facultative anaerobic sugar fermentation, bile tolerance; opportunistic pathogenic and drug resistance risk |
| Bacteria/Bacillota | Erysipelatoclostridium | Genus | Increase | Lipid metabolism modulation, bile acid biotransformation and immune interaction |
| Bacteria/Bacillota | Erysipelotrichaceae incertae sedis | Genus | Increase | Lipid and sterol metabolism, nutrient harvesting and mucosal immune regulation |
| Bacteria/Bacillota | Eubacterium_g23 | Genus | Increase | Dietary fiber degradation, butyrate biosynthesis |
| Bacteria/Bacillota | Eubacterium_sp.CAG：156 | Genus | Increase | Polysaccharide fermentation, butyrate production |
| Bacteria/Bacillota | Eubacterium_sp.CAG：202 | Genus | Increase | Polysaccharide fermentation, butyrate production |
| Bacteria/Bacillota | Gemella | Genus | Increase | Mucosal colonization, carbohydrate fermentation; opportunistic pathogenic potential |
| Bacteria/Bacillota | Holdemanella | Genus | Increase | Fatty acid metabolism, carbohydrate fermentation and immune regulation |
| Bacteria/Bacillota | Holdemania | Genus | Increase | Amino acid fermentation |
| Bacteria/Bacillota | Hungatella | Genus | Increase | Amino acid fermentation |
| Bacteria/Bacillota | Lachnobacterium | Genus | Increase | Dietary fiber fermentation |
| Bacteria/Bacillota | Megasphaera | Genus | Increase | Carbohydrate fermentation, acetate and propionate production |
| Bacteria/Bacillota | Oribacterium | Genus | Increase | Carbohydrate heterofermentation, organic acid synthesis |
| Bacteria/Bacillota | Oscillospira | Genus | Increase | Butyrate production, complex glycan fermentation |
| Bacteria/Bacillota | Paenibacillus | Genus | Increase | Complex polysaccharide degradation |
| Bacteria/Bacillota | Parvimonas | Genus | Increase | Anaerobic proteolytic fermentation; opportunistic pathogenic potential in mixed infections |
| Bacteria/Bacillota | Peptostreptococcus | Genus | Increase | Anaerobic amino acid fermentation, proteolytic metabolism; opportunistic pathogenic potential |
| Bacteria/Bacillota | Ruminococcaceae_UCG-014 | Genus | Increase | Resistant starch degradation |
| Bacteria/Bacillota | Ruminococcus OTU11 | Genus | Increase | Complex polysaccharide degradation |
| Bacteria/Bacillota | Ruthenibacterium | Genus | Increase | Carbohydrate fermentation |
| Bacteria/Bacillota | Sellimonas | Genus | Increase | Dietary glycan fermentation |
| Bacteria/Bacillota | Shuttleworthia_FJ848393 | Genus | Increase | Carbohydrate fermentation |
| Bacteria/Bacillota | Thomasclavelia | Genus | Increase | Lactate utilization, butyrate biosynthesis and anti-inflammation |
| Bacteria/Bacillota | Mediterraneibacter | Genus | Increase | Dietary fiber degradation, butyrate production and mucosal immune homeostasis |
| Bacteria/Bacillota | Blautia glucerasea | Species | Decrease | Carbohydrate fermentation, SCFA synthesis and bile acid biotransformation |
| Bacteria/Bacillota | Blautia luti | Species | Decrease | Carbohydrate fermentation, SCFA synthesis and bile acid biotransformation |
| Bacteria/Bacillota | Blautia provencensis | Species | Decrease | Carbohydrate fermentation, SCFA synthesis and bile acid biotransformation |
| Bacteria/Bacillota | Blautia_obeum | Species | Decrease | Carbohydrate fermentation, SCFA synthesis and bile acid biotransformation |
| Bacteria/Bacillota | Blautia_wexlerae | Species | Decrease | Carbohydrate fermentation, SCFA synthesis and bile acid biotransformation |
| Bacteria/Bacillota | Faecaliclostridium leptum (Clostridium leptum) | Species | Decrease | Dietary fiber fermentation, butyrate biosynthesis and anti-inflammation |
| Bacteria/Bacillota | Pseudocoprococcus catus (Coprococcus catus) | Species | Decrease | Carbohydrate fermentation, butyrate production and immune modulation |
| Bacteria/Bacillota | Allocoprococcus comes (Coprococcus comes) | Species | Decrease | Carbohydrate fermentation, butyrate production and immune modulation |
| Bacteria/Bacillota | Eubacterium eligens | Species | Decrease | Pectin degradation, butyrate biosynthesis and mucosal immune regulation |
| Bacteria/Bacillota | Eubacterium ventriosum | Species | Decrease | Protein and amino acid fermentation, SCFA formation |
| Bacteria/Bacillota | Anaerobutyricum hallii ( Eubacterium hallii ) | Species | Decrease | Lactate utilization, butyrate biosynthesis and metabolic homeostasis |
| Bacteria/Bacillota | Anaerobutyricum hallii CAG:12 ( Eubacterium hallii CAG:12 ) | Species | Decrease | Lactate utilization, butyrate biosynthesis and metabolic homeostasis |
| Bacteria/Bacillota | Faecalibacterium prausnitzii | Species | Decrease | Butyrate biosynthesis, strong anti-inflammation and gut homeostasis |
| Bacteria/Bacillota | Flintibacter butyricus | Species | Decrease | Butyrate production, dietary polysaccharide degradation |
| Bacteria/Bacillota | Fusicatenibacter saccharivorans | Species | Decrease | Butyrate production, anti-inflammatory immune regulation |
| Bacteria/Bacillota | Gemmiger formicilis | Species | Decrease | Carbohydrate fermentation, butyrate synthesis |
| Bacteria/Bacillota | Lacticaseibacillus paracasei Shirota | Species | Decrease | Carbohydrate fermentation, lactic acid production, gut barrier maintenance and immune modulation |
| Bacteria/Bacillota | Lactobacillus acidophilus | Species | Decrease | Carbohydrate fermentation, lactic acid production, gut barrier maintenance and mucosal immune regulation |
| Bacteria/Bacillota | Lactobacillus acidophilus NCIMB 30442 | Species | Decrease | Carbohydrate fermentation, lactic acid production, pathogen inhibition and immune modulation |
| Bacteria/Bacillota | Lactobacillus acidophilus W22 | Species | Decrease | Carbohydrate fermentation, lactic acid production, pathogen inhibition and immune modulation |
| Bacteria/Bacillota | Levilactobacillus brevis (Lactobacillus brevis) | Species | Decrease | Heterolactic fermentation, lactic acid production and immune regulation |
| Bacteria/Bacillota | Lactobacillus bulgaricus | Species | Decrease | Homolactic fermentation, lactic acid biosynthesis and nutrient hydrolysis |
| Bacteria/Bacillota | Lacticaseibacillus casei (Lactobacillus casei) | Species | Decrease | Carbohydrate fermentation, lactic acid production, gut barrier maintenance and immune modulation |
| Bacteria/Bacillota | Lacticaseibacillus casei W56 (Lactobacillus casei W56) | Species | Decrease | Carbohydrate fermentation, lactic acid production, pathogen inhibition and immune modulation |
| Bacteria/Bacillota | Lactobacillus gasseri | Species | Decrease | Carbohydrate fermentation, lactic acid production, cholesterol metabolism modulation |
| Bacteria/Bacillota | Lactobacillus helveticus R0052 | Species | Decrease | Carbohydrate fermentation, lactic acid production, anti-inflammation and immune regulation |
| Bacteria/Bacillota | Lactobacillus helveticus Rosell®-52 | Species | Decrease | Carbohydrate fermentation, lactic acid production, anti-inflammation and immune regulation |
| Bacteria/Bacillota | Lacticaseibacillus lactis W19 (Lactobacillus lactis W19) | Species | Decrease | Carbohydrate fermentation, lactic acid production, pathogen inhibition and immune modulation |
| Bacteria/Bacillota | Lacticaseibacillus paracasei (Lactobacillus paracasei) | Species | Decrease | Carbohydrate fermentation, lactic acid production, gut barrier maintenance and immune modulation |
| Bacteria/Bacillota | Lactobacillus paracasei NCIMB 30439 | Species | Decrease | Carbohydrate fermentation, lactic acid production, pathogen inhibition and immune modulation |
| Bacteria/Bacillota | Lactobacillus paracasei W20 | Species | Decrease | Carbohydrate fermentation, lactic acid production, pathogen inhibition and immune modulation |
| Bacteria/Bacillota | Lactiplantibacillus plantarum (Lactobacillus plantarum) | Species | Decrease | Carbohydrate fermentation, lactic acid production, gut barrier maintenance and immune modulation |
| Bacteria/Bacillota | Lactobacillus plantarum NCIMB 30437 | Species | Decrease | Carbohydrate fermentation, lactic acid production, pathogen inhibition and immune modulation |
| Bacteria/Bacillota | Lactobacillus plantarum W62 | Species | Decrease | Carbohydrate fermentation, lactic acid production, pathogen inhibition and immune modulation |
| Bacteria/Bacillota | Lacticaseibacillus rhamnosus (Lactobacillus rhamnosus) | Species | Decrease | Carbohydrate fermentation, lactic acid production, gut barrier maintenance and immune modulation |
| Bacteria/Bacillota | Lactobacillus rhamnosus R0011 | Species | Decrease | Carbohydrate fermentation, lactic acid production, pathogen inhibition and immune modulation |
| Bacteria/Bacillota | Ligilactobacillus salivarius (Lactobacillus salivarius) | Species | Decrease | Carbohydrate fermentation, lactic acid production, pathogen inhibition and immune regulation |
| Bacteria/Bacillota | Lactobacillus salivarius W24 | Species | Decrease | Carbohydrate fermentation, lactic acid production, pathogen inhibition and immune regulation |
| Bacteria/Bacillota | Limosilactobacillus reuteri (Limosilactobacillus reuteri) | Species | Decrease | Carbohydrate fermentation, lactic acid and reuterin production, pathogen inhibition |
| Bacteria/Bacillota | Pediococcus acidilactici CCFM6432 | Species | Decrease | Carbohydrate fermentation, lactic acid production and antimicrobial activity |
| Bacteria/Bacillota | Phascolarctobacterium succinatutens | Species | Decrease | Succinate utilization, propionate biosynthesis and metabolic regulation |
| Bacteria/Bacillota | Roseburia faecis | Species | Decrease | Butyrate biosynthesis, dietary fiber degradation and anti-inflammation |
| Bacteria/Bacillota | Roseburia hominis | Species | Decrease | Butyrate biosynthesis, dietary fiber degradation and anti-inflammation |
| Bacteria/Bacillota | Roseburia intestinalis | Species | Decrease | Butyrate biosynthesis, dietary fiber degradation and anti-inflammation |
| Bacteria/Bacillota | Ruminococcus bromii | Species | Decrease | Resistant starch degradation, SCFA production and microbial cross-feeding |
| Bacteria/Bacillota | Ruminococcus callidus | Species | Decrease | Complex polysaccharide degradation, SCFA formation |
| Bacteria/Bacillota | Acidaminococcus fermentans DSM 20731 | Species | Increase | Amino acid fermentation, succinate and acetate biosynthesis, cross-feeding with gut microbiota |
| Bacteria/Bacillota | Acidaminococcus_fermentans | Species | Increase | Amino acid fermentation |
| Bacteria/Bacillota | Bacillus cereus | Species | Increase | Facultative anaerobic metabolism, spore formation, toxin biosynthesis; opportunistic pathogenic potential |
| Bacteria/Bacillota | Christensenella timonensis | Species | Increase | Carbohydrate fermentation |
| Bacteria/Bacillota | Clostridium bolteae | Species | Increase | Polysaccharide fermentation, proteolytic metabolism; strain-dependent pro-inflammatory potential |
| Bacteria/Bacillota | Clostridium citroniae | Species | Increase | Carbohydrate fermentation, short-chain fatty acid biosynthesis, gut commensal |
| Bacteria/Bacillota | Clostridium innocuum | Species | Increase | Carbohydrate fermentation, bile resistance; opportunistic pathogenic potential |
| Bacteria/Bacillota | Clostridium porci | Species | Increase | Dietary carbohydrate fermentation |
| Bacteria/Bacillota | Clostridium propionicum | Species | Increase | Amino acid and sugar fermentation, propionate and acetate biosynthesis |
| Bacteria/Bacillota | Clostridium saccharolyticum WM1 | Species | Increase | Anaerobic polysaccharide degradation, ethanol and organic acid biosynthesis |
| Bacteria/Bacillota | Eisenbergiella massiliensis | Species | Increase | Anaerobic carbohydrate fermentation, volatile fatty acid production, gut commensal |
| Bacteria/Bacillota | Fenollaria timonenis | Species | Increase | Anaerobic sugar fermentation |
| Bacteria/Bacillota | Hungatella hathewayi | Species | Increase | Polysaccharide fermentation, SCFA production, bile acid biotransformation |
| Bacteria/Bacillota | Pseudoflavonifractor capillosus | Species | Increase | Flavonoid degradation, carbohydrate fermentation, gut metabolic modulation |
| Bacteria/Bacillota | Ruminococcus torques | Species | Increase | Mucus glycan degradation |
| Bacteria/Bacillota | Streptococcus thermophilus NCIMB 30438 | Species | Increase | Oligosaccharide fermentation |

Table 1 Changes in the relative abundance of gut bacterial taxa from *Bacillota* of Kingdom Bacteria at the family, genus, and species levels in patients with depression

Note: Red indicates increased abundance; green indicates decreased abundance; purple denotes bidirectional changes in abundance.
