# Supplementary material for: Characteristics of gut microbiota dysbiosis in patients with depression: a visual analysis of knowledge maps
Source: Front Microbiol. 2026 Sep 7;17:1937281. doi: 10.3389/fmicb.2026.1937281 (PMC13591385; doi:10.3389/fmicb.2026.1937281)
Supplement: Supplementary file 2 [file Table_2.docx]

| **Taxonomic level** | **Taxon** | **Rank** | ****Gut****  ****microbiota abundance**** | ****Proposed function**** |
| --- | --- | --- | --- | --- |
| Bacteria/Actinomycetota | Actinomycetaceae | Family | Bidirectional | Carbohydrate and protein fermentation, organic acid synthesis; strain-dependent pro-inflammatory potential |
| Bacteria/Actinomycetota | Bifidobacteriaceae | Family | Bidirectional | Oligosaccharide fermentation, acetate production, gut barrier maintenance and mucosal immune regulation |
| Bacteria/Actinomycetota | Corynebacteriaceae | Family | Bidirectional | Amino acid and lipid metabolism, organic acid secretion; mostly commensal, partial opportunistic pathogenicity |
| Bacteria/Actinomycetota | Dermococcaceae | Family | Bidirectional | Aerobic carbohydrate metabolism, amino acid catabolism; predominantly skin and mucosal commensals |
| Bacteria/Actinomycetota | Micromonosporaceae | Family | Bidirectional | Complex polysaccharide degradation, antimicrobial secondary metabolite biosynthesis |
| Bacteria/Actinomycetota | Coriobacteriaceae | Family | Bidirectional | Bile acid biotransformation, steroid metabolism and lipid homeostasis regulation |
| Bacteria/Actinomycetota | Eggerthellaceae | Family | Bidirectional | Cholesterol conversion, bile acid metabolism and host lipid regulation |
| Bacteria/Actinomycetota | Actinomyces | Genus | Increase | Carbohydrate and protein fermentation, organic acid synthesis; strain-dependent invasive pathogenic potential |
| Bacteria/Actinomycetota | Adlercreutzia | Genus | Increase | Bile acid biotransformation, steroid metabolism and host lipid homeostasis modulation |
| Bacteria/Actinomycetota | Coriobacteriineae | Genus | Increase | Bile acid and steroid transformation, aromatic compound metabolism |
| Bacteria/Actinomycetota | Eggerthella | Genus | Increase | Cholesterol conversion, bile acid metabolism; opportunistic pathogenic potential |
| Bacteria/Actinomycetota | Geodermatophilus | Genus | Increase | Aerobic heterotrophic metabolism, stress resistance; predominantly environmental soil bacteria |
| Bacteria/Actinomycetota | Olsenella | Genus | Increase | Carbohydrate fermentation, organic acid production, mucosal commensal |
| Bacteria/Actinomycetota | Pauljensenia | Genus | Increase | Oligosaccharide fermentation, organic acid biosynthesis and immune interaction |
| Bacteria/Actinomycetota | Rothia | Genus | Increase | Mucosal colonization, carbohydrate metabolism; opportunistic pathogenic potential in immunocompromised hosts |
| Bacteria/Actinomycetota | Scardovia | Genus | Increase | Oligosaccharide fermentation, acid production; oral and gastrointestinal commensal |
| Bacteria/Actinomycetota | Collinsella | Genus | Bidirectional | English: Bile acid biotransformation, steroid and cholesterol metabolism, modulation of host lipid homeostasis |
| Bacteria/Actinomycetota | Bifidobacterium adolescentis | Species | Decrease | Oligosaccharide fermentation, acetate production, gut barrier maintenance and immune modulation |
| Bacteria/Actinomycetota | Bifidobacterium animalis | Species | Decrease | Oligosaccharide fermentation, SCFA synthesis, pathogen inhibition and mucosal immune regulation |
| Bacteria/Actinomycetota | Bifidobacterium bifidum | Species | Decrease | Human milk oligosaccharide utilization, acetate production, gut barrier protection and anti-inflammation |
| Bacteria/Actinomycetota | Bifidobacterium bifidum W23 | Species | Decrease | Oligosaccharide fermentation, acetate production, pathogen inhibition and immune modulation |
| Bacteria/Actinomycetota | Bifidobacterium breve | Species | Decrease | Oligosaccharide fermentation, acetate production, metabolic regulation and immune homeostasis |
| Bacteria/Actinomycetota | Bifidobacterium breve NCIMB 30441 | Species | Decrease | Oligosaccharide fermentation, acetate production, pathogen inhibition and immune modulation |
| Bacteria/Actinomycetota | Bifidobacterium infantis | Species | Decrease | Human milk oligosaccharide degradation, acetate biosynthesis and mucosal immune regulation |
| Bacteria/Actinomycetota | Bifidobacterium infantis NCIMB 30436 | Species | Decrease | Oligosaccharide fermentation, acetate production, pathogen inhibition and immune modulation |
| Bacteria/Actinomycetota | Bifidobacterium lactis | Species | Decrease | Oligosaccharide fermentation, SCFA synthesis, pathogen inhibition and immune regulation |
| Bacteria/Actinomycetota | Bifidobacterium lactis W51 | Species | Decrease | Oligosaccharide fermentation, acetate production, pathogen inhibition and immune modulation |
| Bacteria/Actinomycetota | Bifidobacterium lactis W52 | Species | Decrease | Oligosaccharide fermentation, acetate production, pathogen inhibition and immune modulation |
| Bacteria/Actinomycetota | Bifidobacterium longum | Species | Decrease | Complex oligosaccharide fermentation, acetate production, bile acid transformation and immune homeostasis |
| Bacteria/Actinomycetota | Bifidobacterium longum NCIMB 30435 | Species | Decrease | Oligosaccharide fermentation, acetate production, pathogen inhibition and immune modulation |
| Bacteria/Actinomycetota | Bifidobacterium longum R0175 | Species | Decrease | Oligosaccharide fermentation, acetate production, anti-inflammation and immune regulation |
| Bacteria/Actinomycetota | Bifidobacterium longum Rosell®-175 | Species | Decrease | Oligosaccharide fermentation, acetate production, anti-inflammation and immune regulation |
| Bacteria/Actinomycetota | Bifidobacterium longum subsp | Species | Decrease | Complex oligosaccharide fermentation, acetate production and mucosal immune homeostasis |
| Bacteria/Actinomycetota | Corynebacterium aurimucosum | Species | Increase | Mucosal colonization, aromatic amine metabolism, black pigment biosynthesis; opportunistic pathogenic potential |
| Bacteria/Actinomycetota | Saccharomonospora viridis | Species | Increase | Thermophilic polysaccharide degradation, thermostable amylase/protease secretion, antimicrobial metabolite biosynthesis |
| Bacteria/Bacteroidota | Bacteroidaceae | Family | Bidirectional | Complex polysaccharide degradation, SCFA biosynthesis and bile acid biotransformation |
| Bacteria/Bacteroidota | Barnesiellaceae | Family | Bidirectional | Dietary glycan fermentation, SCFA production and immune modulation |
| Bacteria/Bacteroidota | Marinilabiliaceae | Family | Bidirectional | Anaerobic polysaccharide hydrolysis, organic acid synthesis; mostly environmental microbes |
| Bacteria/Bacteroidota | Muribaculaceae | Family | Bidirectional | Dietary fiber degradation, propionate biosynthesis and gut metabolic regulation |
| Bacteria/Bacteroidota | Porphyromonadaceae | Family | Bidirectional | Proteolytic fermentation, amino acid metabolism; strain-dependent pro-inflammatory effects |
| Bacteria/Bacteroidota | Prevotellaceae | Family | Bidirectional | Hemicellulose degradation, acetate and propionate production, carbohydrate fermentation |
| Bacteria/Bacteroidota | Rikenellaceae | Family | Bidirectional | Complex glycan degradation, bile acid transformation and lipid metabolism regulation |
| Bacteria/Bacteroidota | Tannerellaceae | Family | Bidirectional | Polysaccharide fermentation, SCFA synthesis and host immune interaction |
| Bacteria/Bacteroidota | Flavobacteriaceae | Family | Decrease | polysaccharide degradation, proteolysis |
| Bacteria/Bacteroidota | Alloprevotella | Genus | Decrease | Carbohydrate fermentation, acetate and succinate production, immune modulation |
| Bacteria/Bacteroidota | Bacteroides dorei OTU4 | Genus | Decrease | Complex polysaccharide degradation, SCFA synthesis and bile acid biotransformation |
| Bacteria/Bacteroidota | Bacteroidetes bacteria | Genus | Decrease | Glycan degradation, SCFA biosynthesis and nutrient metabolism |
| Bacteria/Bacteroidota | Barnesiella | Genus | Decrease | Dietary glycan fermentation, SCFA production and anti-inflammation |
| Bacteria/Bacteroidota | Butyricimonas | Genus | Decrease | Butyrate biosynthesis, polysaccharide degradation and gut barrier protection |
| Bacteria/Bacteroidota | Capnocytophaga | Genus | Decrease | Proteolytic metabolism, carbohydrate fermentation; mucosal commensal, opportunistic pathogen |
| Bacteria/Bacteroidota | Macellibacteroides | Genus | Decrease | Amino acid fermentation, proteolysis and organic acid formation |
| Bacteria/Bacteroidota | Phocaeicola | Genus | Decrease | Amino acid fermentation, proteolysis and organic acid formation |
| Bacteria/Bacteroidota | Prevotella_2 | Genus | Decrease | Hemicellulose fermentation, acetate and propionate production |
| Bacteria/Bacteroidota | Prevotella_9 | Genus | Decrease | Hemicellulose fermentation, acetate and propionate production |
| Bacteria/Bacteroidota | Prevotellaceae NK3B31 group | Genus | Decrease | Carbohydrate fermentation, short-chain fatty acid synthesis and metabolic regulation |
| Bacteria/Bacteroidota | Bacteroides | Genus | Bidirectional | Complex polysaccharide degradation, SCFA biosynthesis and bile acid biotransformation |
| Bacteria/Bacteroidota | Alistipes | Genus | Bidirectional | Bile acid transformation, complex glycan degradation and immune modulation |
| Bacteria/Bacteroidota | Parabacteroides | Genus | Bidirectional | Polysaccharide fermentation, succinate/propionate production and metabolic homeostasis |
| Bacteria/Bacteroidota | Prevotella | Genus | Bidirectional | Hemicellulose degradation, acetate and propionate biosynthesis, carbohydrate fermentation |
| Bacteria/Bacteroidota | Bacteroides dorei OTU6 | Genus | Increase | Complex polysaccharide degradation, SCFA biosynthesis and bile acid biotransformation |
| Bacteria/Bacteroidota | Haliscomenobacter | Genus | Increase | Anaerobic polysaccharide hydrolysis, filamentous growth; mostly environmental sludge bacteria |
| Bacteria/Bacteroidota | Paramuribaculum | Genus | Increase | Dietary fiber degradation, propionate biosynthesis and gut metabolic regulatio |
| Bacteria/Bacteroidota | Paraprevotella | Genus | Increase | Carbohydrate fermentation, acetate and succinate production, immune modulation |
| Bacteria/Bacteroidota | Porphyromonas | Genus | Increase | Proteolytic fermentation, amino acid metabolism; strain-dependent pro-inflammatory pathogenic potential |
| Bacteria/Bacteroidota | Alistipes shahii | Species | Decrease | Complex glycan degradation, bile acid biotransformation and immune modulation |
| Bacteria/Bacteroidota | Bacteroides barnesiae | Species | Decrease | Complex polysaccharide degradation, SCFA biosynthesis and bile acid transformation |
| Bacteria/Bacteroidota | Bacteroides massillensis | Species | Decrease | Dietary glycan fermentation, SCFA production and nutrient metabolism |
| Bacteria/Bacteroidota | Helicobacter felis ATCC 49179 | Species | Decrease | Gastric mucosal colonization, urea hydrolysis, strain-dependent pro-inflammatory effects |
| Bacteria/Bacteroidota | Alistipes obesi | Species | Increase | Complex glycan degradation, bile acid biotransformation and lipid metabolism modulation |
| Bacteria/Pseudomonadota | Reyranellaceae | Family | Increase | Aerobic heterotrophic metabolism, organic substrate degradation |
| Bacteria/Pseudomonadota | Sphingomonadaceae | Family | Increase | Aromatic compound degradation, sphingolipid biosynthesis; mostly environmental commensals |
| Bacteria/Pseudomonadota | Oxalobacteraceae | Family | Increase | Oxalate degradation, organic acid metabolism and gut ion homeostasis regulation |
| Bacteria/Pseudomonadota | Burkholderiaceae | Family | Increase | Heterotrophic metabolism, bioactive secondary metabolite synthesis; strain-dependent invasive pathogenic potential |
| Bacteria/Pseudomonadota | Enterobacteriaceae | Family | Increase | Facultative anaerobic carbohydrate fermentation |
| Bacteria/Pseudomonadota | Moraxellaceae | Family | Increase | Aerobic carbohydrate catabolism, mucosal colonization; opportunistic pathogenic and drug resistance risk |
| Bacteria/Pseudomonadota | Sutterellaceae | Family | Decrease | Carbohydrate fermentation, organic acid production and mucosal immune modulation |
| Bacteria/Pseudomonadota | Pasteurellaceae | Family | Decrease | Carbohydrate metabolism, proteolysis; mucosal commensal with opportunistic pathogenic potential |
| Bacteria/Pseudomonadota | Succinivibrionaceae | Family | Decrease | Carbohydrate fermentation, succinate and acetate biosynthesis, gut metabolic regulation |
| Bacteria/Pseudomonadota | Aphodousia | Genus | Decrease | Anaerobic glycan fermentation, short-chain organic acid synthesis |
| Bacteria/Pseudomonadota | Avibacterium | Genus | Decrease | Carbohydrate fermentation; animal mucosal commensal, opportunistic pathogen |
| Bacteria/Pseudomonadota | Bordetella | Genus | Decrease | Mucosal adhesion, virulence factor secretion, pro-inflammatory immune activation |
| Bacteria/Pseudomonadota | Brucella | Genus | Decrease | Intracellular colonization, host immune interference, pathogenic phenotype |
| Bacteria/Pseudomonadota | Citrobacter | Genus | Decrease | Carbohydrate fermentation, citrate utilization; opportunistic pathogenic potential |
| Bacteria/Pseudomonadota | Oxalobacter | Genus | Decrease | Oxalate degradation, organic acid metabolism and gut ion homeostasis |
| Bacteria/Pseudomonadota | Polymorphobacter | Genus | Decrease | Aerobic carbohydrate catabolism, environmental polysaccharide degradation |
| Bacteria/Pseudomonadota | Pseudomonas | Genus | Decrease | Aerobic heterotrophic metabolism, antimicrobial metabolite synthesis; opportunistic pathogen |
| Bacteria/Pseudomonadota | Solisphingomonas | Genus | Decrease | Aromatic compound degradation, aerobic carbohydrate metabolism; environmental bacterium |
| Bacteria/Pseudomonadota | Succinivibrio | Genus | Decrease | Carbohydrate fermentation, succinate production and cross-feeding with gut microbes |
| Bacteria/Pseudomonadota | Sutterella | Genus | Decrease | Carbohydrate fermentation, organic acid secretion and immune interaction |
| Bacteria/Pseudomonadota | Enterobacter | Genus | Bidirectional | Carbohydrate fermentation, facultative anaerobic metabolism; opportunistic pathogenic risk |
| Bacteria/Pseudomonadota | Parasutterella | Genus | Bidirectional | Carbohydrate fermentation, succinate synthesis and host metabolic modulation |
| Bacteria/Pseudomonadota | Aquisphaera | Genus | Increase | Aerobic heterotrophic metabolism, environmental polysaccharide degradation |
| Bacteria/Pseudomonadota | Acinetobacter | Genus | Increase | Aerobic carbohydrate catabolism; opportunistic pathogenic potential and drug resistance risk |
| Bacteria/Pseudomonadota | Burkholderia | Genus | Increase | Heterotrophic metabolism, secondary metabolite synthesis; strain-dependent invasive pathogenicity |
| Bacteria/Pseudomonadota | Enterobacter_CP017181_s | Genus | Increase | Carbohydrate fermentation, facultative anaerobic metabolism; opportunistic pathogenic risk |
| Bacteria/Pseudomonadota | Erythrobacter | Genus | Increase | Aerobic photoheterotrophic metabolism, carotenoid biosynthesis; mainly environmental bacteria |
| Bacteria/Pseudomonadota | Escherichia | Genus | Increase | Carbohydrate fermentation, facultative anaerobic growth; strain-dependent pathogenic potential |
| Bacteria/Pseudomonadota | Haemophilus | Genus | Increase | Mucosal colonization, heme-dependent metabolism; opportunistic pathogenic potential |
| Bacteria/Pseudomonadota | Klebsiella | Genus | Increase | Carbohydrate fermentation, capsular polysaccharide synthesis; opportunistic pathogenic risk |
| Bacteria/Pseudomonadota | Massilia | Genus | Increase | Aerobic heterotrophic metabolism, organic compound degradation; mostly environmental microbes |
| Bacteria/Pseudomonadota | Ruminobacter | Genus | Increase | Carbohydrate fermentation, succinate production, rumen fiber degradation |
| Bacteria/Pseudomonadota | Shigella | Genus | Increase | Mucosal invasion, virulence factor secretion, pro-inflammatory pathogenic phenotype |
| Bacteria/Pseudomonadota | Pseudescherichia | Genus | Increase | Carbohydrate fermentation, facultative anaerobic metabolism |
| Bacteria/Pseudomonadota | Pelomonas | Genus | Increase | Aerobic heterotrophic metabolism, aromatic compound degradation |
| Bacteria/Pseudomonadota | Escherichia coli | Species | Increase | Carbohydrate fermentation, vitamin biosynthesis; strain-dependent commensal or pathogenic phenotype |
| Bacteria/Pseudomonadota | Escherichia-Shigella | Species | Increase | Carbohydrate fermentation, mucosal invasion |
| Bacteria/Verrucomicrobiota | Akkermansiaceae | Family | Bidirectional | Mucin glycan degradation, gut barrier regulation and host metabolic modulation |
| Bacteria/Verrucomicrobiota | Terrimicrobiaceae | Family | Increase |  |
| Bacteria/Verrucomicrobiota | Victivallis | Genus | Decrease | Anaerobic carbohydrate fermentation, organic acid biosynthesis |
| Bacteria/Verrucomicrobiota | Akkermansia | Genus | Bidirectional | Mucin glycan degradation, gut barrier maintenance and host metabolic regulation |
| Bacteria/Synergistota | Pyramidobacter | Genus | Decrease | Amino acid fermentation, proteolytic metabolism and cross-feeding |
| Bacteria/Fusobacteriota | Fusobacteriaceae | Family | Increase | Amino acid fermentation, proteolytic metabolism; strain-dependent pro-inflammatory and pathogenic potential |
| Bacteria/Fusobacteriota | Fusobacterium | Genus | Bidirectional | Amino acid fermentation, proteolytic metabolism; strain-dependent pro-inflammatory potential |
| Bacteria/Desulfobacterota | Desulfovibrionales | Family | Bidirectional | Sulfate reduction, hydrogen metabolism, sulfide production and immune interaction |
| Bacteria/Desulfobacterota | Bilophila | Genus | Increase | Bile salt resistance, amino acid fermentation, sulfide production and immune interaction |
| Bacteria/Desulfobacterota | Desulfovibrio | Genus | Increase | Sulfate reduction, hydrogen metabolism, sulfide biosynthesis and gut barrier modulation |
| Bacteria/Deinococcota | Deinococcaceae | Family | Increase | Radiation and oxidative stress resistance |
| Bacteria/Deinococcota | Deinococcus | Genus | Increase | Extreme stress tolerance, aerobic carbohydrate catabolism, DNA damage repair capacity |
| Bacteria/Campylobacterota | Campylobacteraceae | Family | Increase | Mucosal colonization, amino acid fermentation; strain-dependent pro-inflammatory and pathogenic potential |
| Bacteria/Campylobacterota | Mesophilic Helicobacter | Genus | Increase | Gastric mucosal adhesion, urea hydrolysis, immune activation and pro-inflammatory effects |
| Bacteria/Actinobacteria | Actinomycineae | Family | Increase | Carbohydrate and protein fermentation, organic acid synthesis; strain-dependent pro-inflammatory risk |

Table 2 Alterations in the relative abundance of gut bacterial taxa derived from other phyla of Kingdom Bacteria at the family, genus, and species levels in patients with major depressive disorde

Note: Red indicates increased abundance; green indicates decreased abundance; purple denotes bidirectional changes in abundance.
