# Supplementary material for: Characteristics of gut microbiota dysbiosis in patients with depression: a visual analysis of knowledge maps
Source: Front Microbiol. 2026 Sep 7;17:1937281. doi: 10.3389/fmicb.2026.1937281 (PMC13591385; doi:10.3389/fmicb.2026.1937281)
Supplement: Supplementary file 3 [file Table_3.docx]

| Fungi/Ascomycota | Neocosmospora | Genus | Increase | Plant parasitism, lignocellulose degradation, mycotoxin biosynthesis; opportunistic invasive infection potential |
| --- | --- | --- | --- | --- |
| Fungi/Ascomycota | Chaetomium | Genus | Increase | Lignocellulose decomposition, cellulase secretion; strain-dependent invasive pathogenic potential |
| Fungi/Ascomycota | Cercospora beticola | Species | Decrease | Phytopathogenic metabolism, lignocellulose degradation |
| Fungi/Ascomycota | Botrytis cinerea | Species | Decrease | Saprotrophic polysaccharide hydrolysis |
| Fungi/Ascomycota | Saccharomyces cerevisiae boulardii | Species | Decrease | Anaerobic carbohydrate fermentation, pathogen inhibition, gut barrier protection and immune modulation |
| Fungi/Ascomycota | Talaromyces marneffei | Species | Increase | Dimorphic fungal metabolism, intracellular survival, invasive pathogenic potential in immunocompromised individuals |
| Fungi/Basidiomycota | Occultifur | Species | Increase | Aerobic carbohydrate catabolism, saprotrophic growth; mostly environmental commensal |
| Fungi/Basidiomycota | Apiotrichum | Species | Increase | Carbohydrate fermentation, lipid metabolism |

Table 3 Changes in the relative abundance of gut fungal taxa at the family, genus, and species levels in patients with depression

Note: Red indicates increased abundance; green indicates decreased abundance; purple denotes bidirectional changes in abundance.
